# Multidimensional phenotyping of the post‐COVID‐19 syndrome: A Swiss survey study

**DOI:** 10.1111/cns.13938

**Published:** 2022-08-16

**Authors:** Lara Diem, Anina Schwarzwald, Christoph Friedli, Helly Hammer, Livia Gomes‐Fregolente, Jan Warncke, Lea Weber, Nicole Kamber, Andrew Chan, Claudio Bassetti, Anke Salmen, Robert Hoepner

**Affiliations:** ^1^ Department of Neurology Inselspital, Bern University Hospital and University of Bern Bern Switzerland; ^2^ Graduate School for Health Sciences University of Bern Bern Switzerland

**Keywords:** coronavirus, long‐term symptoms, neuropsychiatric symptoms, post‐infectious, SARS‐CoV‐2, viral infection

## Abstract

**Introduction:**

Post‐COVID‐19 syndrome affects approximately 10–25% of people after a COVID‐19 infection, irrespective of initial COVID‐19 severity. The aim of this project was to assess the clinical characteristics, course, and prognosis of post‐COVID‐19 syndrome using a systematic multidimensional approach.

**Patients and Methods:**

An online survey of people with suspected and confirmed COVID‐19 and post‐COVID‐19 syndrome, distributed via Swiss COVID‐19 support groups, social media, and our post‐COVID‐19 consultation, was performed. A total of 8 post‐infectious domains were assessed with 120 questions.

Data were collected from October 15 to December 12, 2021, and 309 participants were included. Analysis of clinical phenomenology of post‐COVID‐19 syndrome was performed using comparative statistics.

**Results:**

The three most prevalent post‐COVID‐19 symptoms in our survey cohort were fatigue (288/309, 93.2%), pain including headache (218/309, 70.6%), and sleep–wake disturbances (mainly insomnia and excessive daytime sleepiness, 145/309, 46.9%). Post‐COVID‐19 syndrome had an impact on work ability, as more than half of the respondents (168/268, 62.7%) reported an inability to work, which lasted on average 26.6 weeks (95% CI 23.5–29.6, range 1–94, *n* = 168). Quality of life measured by WHO‐5 Well‐being Index was overall low in respondents with post‐COVID‐19 syndrome (mean, 95% CI 9.1 [8.5–9.8], range 1–25, *n* = 239).

**Conclusion:**

Fatigue, pain, and sleep–wake disturbances were the main symptoms of the post‐COVID‐19 syndrome in our cohort and had an impact on the quality of life and ability to work in a majority of patients. However, survey respondents reported a significant reduction in symptoms over 12 months. Post‐COVID‐19 syndrome remains a significant challenge. Further studies to characterize this syndrome and to explore therapeutic options are therefore urgently needed.

## Graphical abstract

Fatigue, pain, and sleep–wake disturbances were the main symptoms of the post‐COVID‐19 syndrome in our cohort and had an impact on quality of life and ability to work in a majority of patients. However, survey respondents reported a significant reduction in symptoms over 12 months.

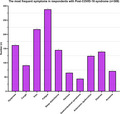

## 1 INTRODUCTION

The SARS‐CoV‐2 pandemic including its post‐infectious sequelae are a major health concern. Post‐COVID‐19 syndrome (also termed Long‐COVID‐19 or Long Haulers) is defined by the World Health Organization (WHO) as follows: “Post COVID‐19 condition occurs in individuals with a history of probable or confirmed SARS‐CoV‐2 infection, usually 3 months from the onset of COVID‐19 with symptoms and that last for at least 2 months and cannot be explained by an alternative diagnosis”. [1] In the year 2021, 204.98 million people have been infected worldwide. [2] Not all of the infected people recover completely, as 9.9–22.1% remain symptomatic even months after the initial COVID‐19 infection [3] and subsequently develop a post‐COVID‐19 syndrome. By extrapolation of these numbers, between 20.29 and 45.30 million people worldwide might have been affected by the post‐COVID‐19 syndrome in 2021. Considering a population of 7.9 billion people [4], a worldwide incidence of the post‐COVID‐19 syndrome between 257–573 per 100.000 can be estimated. Due to the recent pandemic wave of infections with the Omicron variant, which is described to have a threefold higher reproduction rate compared with the Delta variant, numbers of the post‐COVID‐19 syndrome might further increase. [5]

Despite the large amount of affected people and even though post‐infectious syndromes have been described since decades, for example, as the von Economo's syndrome being a post‐encephalitis syndrome [6], our knowledge on post‐infectious fatigue syndrome, in general, is still limited. Post‐COVID‐19 syndrome is characterized by multiple symptoms including fatigue, pain, and sleep disturbances. [7] Recent articles have unequivocally highlighted that fatigue is one of the most frequent symptoms occurring in 30–90% of the affected. [7, 8, 9, 10, 11] Fatigue is defined as a debilitating feeling of mental and/or physical loss of energy and can be accompanied especially in the post‐COVID‐19 syndrome by a post‐exertional malaise. [12]

Post‐COVID‐19 syndrome affects the social well‐being. Studies on non‐hospitalized patients with COVID‐19 showed that between 12 and 23% remain absent from work even 3–7 months after COVID‐19. [11] So far, it is unclear how many of them will remain unable to work and eventually need invalidity pensions. The therapy of the post‐COVID‐19 syndrome remains challenging. The National Institute for Health and Care Excellence (NICE) recommends self‐management and support, including cognitive behavioral therapy (CBT) and graded exercise therapy (GET) [13]. Furthermore, the course of post‐COVID‐19 syndrome is still incompletely detailed. Davis et al. report that 65.2% persons with post‐COVID‐19 syndrome experienced primarily neuropsychiatric symptoms including fatigue for at least 6 months. [7]

In this article, we report results from an online patient survey performed in Switzerland between October 2021 and December 2021. The survey investigated multidimensional aspects of the post‐COVID‐19 syndrome reported by the patients, including demographic characteristics, acute COVID‐19 illness, post‐COVID‐19 symptoms with a special focus on fatigue and sleep disturbances, treatment strategies including vaccination, as well as psychosocial aspects.

## 2 PATIENTS AND METHODS

We conducted an online survey, launched via Survey Monkey (https://de.surveymonkey.com/) on October 15, 2021 to investigate people with post‐COVID‐19 syndrome in Switzerland. The survey was distributed via Swiss COVID‐19 support groups (e.g., Altea Long‐COVID Network [14]), social media (e.g., Twitter and Facebook), and our post‐COVID‐19 consultation at the neurological outpatient department of the University Hospital Bern, Inselspital, Bern, Switzerland. Data were collected from October 15, 2021, to December 12, 2021. All respondents gave digital informed consent prior to participation to our anonymous survey. The Ethics Committee of the Canton of Bern has been informed about the project. It assessed the project as not requiring ethical approval, since the project did not fall under the Swiss Human Research Act, Art. 2, Para. 1. (Req‐2021‐00750). The survey was created in German and translated into the other Swiss national languages French and Italian by Swiss native bilingual speakers. The survey “Conditions of participation” stated in the appropriate language for the patient: “You may participate in the study if the following apply to you: (1) COVID‐19 acute illness occurred at least 12 weeks (3 months) ago. (2) You are at least 18 years old and have a sufficiently good knowledge of German/French/Italian to be able to complete this questionnaire.”

The survey consisted of 120 questions and 60 minutes were needed for completion. To account for post‐COVID‐19 symptoms that can limit sustained focus and attention span, respondents were encouraged to take breaks while completing the survey if necessary. Our survey investigated the following 8 domains: demographic characteristics (24/120 questions), acute COVID‐19 illness (10/120 questions), post‐COVID‐19 symptoms at 5 time points (1–3; 4–6; 7–9; 10–12; >12 months; 11/120 questions), fatigue in post‐COVID‐19 syndrome (22/120 questions), sleep–wake disturbances in post‐COVID‐19 syndrome (10/120 questions), mental disturbances, and social aspects in post‐COVID‐19 syndrome (16/120 questions), therapy of post‐COVID‐19 syndrome (18/120 questions), and vaccination (9/120 questions). In the survey, we integrated the following freely accessible and previously validated scales/indices: Fatigue Severity Scale (FSS) [15], Epworth Sleepiness Scale (ESS) [16], Insomnia Severity Index (ISI) [17], and WHO‐5 Well‐being Index (WHO‐5) [18].

**FIGURE 1:**
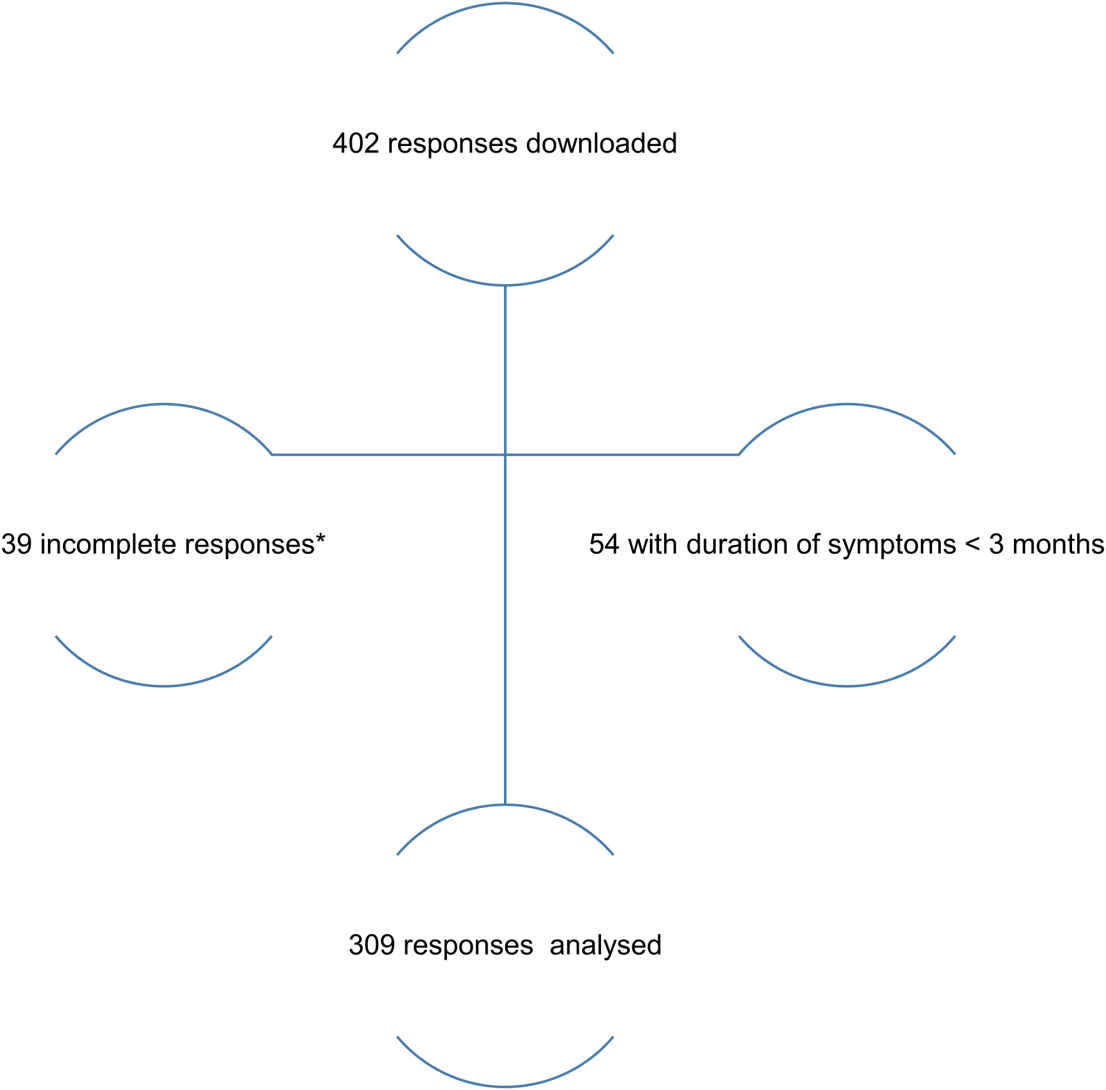
Study flowchart of responses downloaded from the Survey Monkey server on December 12, 2021 *defined as more than 50% missing answers.

In total, 402 anonymized responses were downloaded from the Survey Monkey server on December 12, 2021. 39 incomplete responses, defined as more than 50% missing answers, and 54 responses not fulfilling the WHO post‐COVID‐19 criteria due to symptom duration <3 months were removed, leading to a data set of 309 responses. (Figure 1) Data are presented as mean with 95% confidence interval (95% CI), comparative statistics were used, and type of testing is referred prior to each *p*‐value. A *p*‐value of 0.05 was assumed as significant. In case of multiple testing, a Bonferroni correction was run.

Data sharing statement: Following an open data approach, anonymized data of the cohort can be requested via the corresponding author.

## 3 RESULTS

### 3.1 Basic cohort characteristics

The majority of respondents to our online survey were female (249/309 [80.6%]), and the mean age was 44.6 years (95% Confidence interval [95% CI] 43.4–46.0 years, range 19.0–83.0 years, *n* = 309). The respondents filled out the questionnaire 13.0 months (mean, 95% CI 12.6–13.6 months, range 0.2–24.5 years, *n* = 309) after acute COVID‐19 infection. SARS‐CoV‐2 testing (PCR or antigen test) to confirm initial COVID‐19 was reported as positive in 82.8% (256/309). The most frequent previous disease was bronchial asthma (34/309, 11.0%). Characteristics of the cohort are given in Table 1. The three most prevalent post‐COVID‐19 symptoms in our Swiss cohort were fatigue (288/309, 93.2%), pain including headache (218/309, 70.6%), and sleep disturbances (145/309, 46.9%).

**TABLE 1:** Characteristics of population and of patients with post‐COVID‐19 syndrome

Variable		*n*
Populations characteristics		
Age, years, mean (95% CI)	44.6 (43.4–46.0)	309
Female, *n* (%)	249 (80.6)	309
Nationality		309
Swiss	294 (95.1)	309
German	6 (1.9)	309
French	6 (1.9)	309
Belgian	2 (0.6)	309
Austrian	1 (0.3)	309
Cantons of Switzerland		
Aargau	36	295
Appenzell A.Rh/ Appenzell I.Rh.	0	295
Basel‐Landschaft	16	295
Basel‐Stadt	12	295
Bern	61	295
Freiburg	7	295
Genf	0	295
Glarus	0	295
Graubünden	2	295
Jura	0	295
Luzern	24	295
Neuenburg	2	295
Nidwalden	0	295
Obwalden	1	295
Sankt Gallen	2	295
Schaffhausen	0	295
Schwyz	7	295
Solothurn	11	295
Tessin	2	295
Thurgau	1	295
Uri	1	295
Waadt	10	295
Wallis	3	295
Zug	0	295
Zürich	97	295
Education ≥13 years*, *n* (%)	184 (59.5)	309
Self‐employment, *n* (%)	37 (12.0)	309
Time between onset of acute infection and questionnaire, months, mean (95% CI)	13.0 (12.6–13.6)	299
Symptoms of acute COVID‐19, *n* (%)		
Headache	230 (74.4)	309
Fever	152 (49.2)	309
Anosmia	89 (28.8)	309
Dyspnea	133 (43.0)	309
Cough	178 (57.6)	309
Cold	106 (34.3)	309
Sore throat	144 (46.6)	309
Pain	222 (71.8)	309
Gastrointestinal symptoms	73 (23.6)	309
Fatigue	273 (88.3)	309
Sleep disturbance	103 (33.3)	309
Dizziness	26 (8.4)	309
Skin alteration	26 (8.4)	309
Hospitalization, *n* (%)	33 (10.7)	309
Intubation, *n* (%)	6 (2.0)	309
Oxygen requirement, *n* (%)	29 (9.4)	309
ICU without intubation, *n* (%)	8 (2.6)	309
Positive PCR/Antigen Test, *n* (%)	256 (82.8)	309
Comorbidities, *n* (%)	112 (36.2)	309
Hypertension, *n* (%)	23 (7.4)	309
Diabetes mellitus, *n* (%)	2 (0.6)	309
Depression, *n* (%)	14 (4.5)	309
Hypothyroidism, *n* (%)	17 (5.5)	309
Pain syndrome, *n* (%)	10 (3.2)	309
Asthma bronchiale, *n* (%)	34 (11.0)	309
Cancer, *n* (%)	4 (1.3)	309
Endometriosis, *n* (%)	4 (1.3)	309
Post‐COVID‐19 Syndrome symptoms		
Persistence of symptoms over 3 months, *n* (%)	309 (94.2)	328
Symptoms 1–3 months		
Dyspnea, *n* (%)	162 (52.4)	309
Cough, *n* (%)	91 (29.4)	309
Pain incl. headache, *n* (%)	218 (70.6)	309
Fatigue, *n* (%)	288 (93.2)	309
Sleep disturbance, *n* (%)	145 (46.9)	309
Hair loss, *n* (%)	65 (21.0)	309
Gastrointestinal symptoms, *n* (%)	44 (14.2)	309
Autonomic dysfunction, *n* (%)	124 (40.1)	309
Dizziness, *n* (%)	139 (45.0)	309
Anosmia, *n* (%)	71 (23.0)	309
Symptoms 4–6 months		
Dyspnea, *n* (%)	113 (38.7)	292
Cough, *n* (%)	41 (14.0)	292
Pain incl. headache, *n* (%)	193 (66.1)	292
Fatigue, *n* (%)	251 (86.0)	292
Sleep disturbance, *n* (%)	131 (44.9)	292
Hair loss, *n* (%)	62 (21.2)	292
Gastrointestinal symptoms, *n* (%)	17 (5.8)	292
Autonomic dysfunction, *n* (%)	106 (36.3)	292
Dizziness, *n* (%)	122 (41.8)	292
Anosmia, *n* (%)	56 (19.2)	292
No symptoms	16 (5.5)	292
Symptoms 7–9 months		
Dyspnea, *n* (%)	86 (30.3)	284
Cough, *n* (%)	32 (11.3)	284
Pain incl. headache, *n* (%)	170 (59.9)	284
Fatigue, *n* (%)	217 (76.4)	284
Sleep disturbance, *n* (%)	111 (39.1)	284
Hair loss, *n* (%)	38 (13.4)	284
Gastrointestinal symptoms, *n* (%)	14 (4.9)	284
Autonomic dysfunction, *n* (%)	100 (35.2)	284
Dizziness, *n* (%)	94 (33.1)	284
Anosmia, *n* (%)	55 (19.4)	284
No symptoms	41 (14.4)	284
Symptoms 10–12 months		
Dyspnea, *n* (%)	56 (23.6)	237
Cough, *n* (%)	22 (9.3)	237
Pain incl. headache, *n* (%)	105 (44.3)	237
Fatigue, *n* (%)	147 (62.0)	237
Sleep disturbance, *n* (%)	76 (32.1)	237
Hair loss, *n* (%)	28 (11.8)	237
Gastrointestinal symptoms, *n* (%)	5 (2.1)	237
Autonomic dysfunction, *n* (%)	64 (27.0)	237
Dizziness, *n* (%)	68 (28.7)	237
Anosmia, *n* (%)	34 (14.3)	237
No symptoms	68 (28.7)	237
Symptoms over 12 months		
Dyspnea, *n* (%)	35 (18.1)	193
Cough, *n* (%)	16 (8.3)	193
Pain incl. headache, *n* (%)	45 (23.3)	193
Fatigue, *n* (%)	71 (36.8)	193
Sleep disturbance, *n* (%)	34 (17.6)	193
Hair loss, *n* (%)	10 (5.2)	193
Gastrointestinal symptoms, *n* (%)	3 (1.6)	193
Autonomic dysfunction, *n* (%)	31 (16.1)	193
Dizziness, *n* (%)	29 (15.0)	193
Anosmia, *n* (%)	11 (5.7)	193
No symptoms	113 (58.5)	193
Fatigue in post‐COVID‐19 syndrome		
Fatigue character		
Motor, *n* (%)	32 (12.9)	249
Cognition, *n* (%)	13 (5.2)	249
Both, *n* (%)	204 (81.9)	249
Peak fatigue period		
Morning, *n* (%)	35 (14.6)	240
Afternoon, *n* (%)	100 (41.7)	240
Evening, *n* (%)	63 (26.3)	240
All days, *n* (%)	42 (17.5)	240
FSS, mean (95% CI)	5.7 (5.5–5.9)	255
Sleep disturbances in post‐COVID‐19 syndrome		
Sleep disturbances, *n* (%)	148 (50.3)	294
Insomnia Severity Index, mean (95% CI)	13.3 (12.4–14.1)	241
ESS, mean (95% CI)	10.9 (10.3–11.5)	224
Mental and social condition in post‐COVID‐19 syndrome		
Mood		
Good, *n* (%)	212 (70.0)	303
Moderate, *n* (%)	44 (14.5)	303
Severe, *n* (%)	47 (15.5)	303
WHO‐5 Well‐being Index, mean (95% CI)	9.1 (8.5–9.8)	239
Incapacity to work, *n* (%)	168 (62.7)	268
Duration of incapacity to work, weeks, mean (95% CI)	26.6 (23.5–29.6)	168
Maximal incapacity to work, mean (95% CI)	89.1 (85.5–92.7)	168
Limitations in everyday life, *n* (%)	226 (84.3)	268
Sense of comprehension, *n* (%)	106 (41.1)	258
Therapy/Care of post‐COVID‐19 syndrome		
People with doctor's visit, *n* (%)	269 (87.1)	309
Number of consulted doctors, mean (95% CI)	3.5 (3.2–3.8)	299
General practitioner	266 (86.1)	309
Neurologist	97 (31.4)	309
Pneumologist	118 (38.2)	309
Rheumatologist	17 (5.5)	309
Psychiatrist	61 (19.7)	309
Gastroenterologist	16 (5.2)	309
Dermatologist	22 (7.1)	309
Pain specialist	8 (2.6)	309
Cardiologist	96 (31.1)	309
Psychologist	52 (16.8)	309
Sleep specialist	15 (4.9)	309
Otolaryngologist	43 (13.9)	309
Therapy		
Therapy	225 (72.8)	309
Physiotherapy	112 (36.2)	309
Session per week, mean (95% CI)	1.4 (1.3–1.5)	105
Improvement in %, mean (95% CI)	31.5 (25.9–37.1)	104
Occupational therapy	60 (19.4)	309
Session per week, mean (95% CI)	1.3 (1.0–1.5)	60
Improvement in %, mean (95% CI)	26.5 (19.4–33.6)	58
Relaxation methods	53 (17.2)	309
Session per week, mean (95% CI)	3.0 (2.3–3.6)	45
Improvement in %, mean (95% CI)	35.8 (28.2–43.4)	45
Massage	45 (14.6)	309
Session per week, mean (95% CI)	1.3 (1.1–1.5)	45
Improvement in %, mean (95% CI)	31.3 (23.3–39.1)	44
Rehabilitation	54 (17.4)	309
Duration in weeks, mean (95% CI)	4.9 (4.2–5.7)	54
Improvement in %, mean (95% CI)	30.6 (23.8–37.3)	48
Medication	92 (29.8)	309
Antihistaminic	27 (29.3)	92
Improvement in %, mean (95% CI)	58.1 (49.4–66.8)	26
Vitamins	35 (38.0)	92
Improvement in %, mean (95% CI)	24.2 (15.8–32.7)	33
Antidepressants	22 (23.9)	92
Improvement in %, mean (95% CI)	54.7 (41.6–67.8)	18
Inhalation medicines	23 (25.0)	92
Improvement in %, mean (95% CI)	52.5 (42.4–62.6)	22
Homeopathy	31 (33.7)	92
Improvement in %, mean (95% CI)	14.3 (6.8–21.9)	30
Medications with effect on coagulation	5 (5.4)	92
Steroids	5 (5.4)	92
Blood pressure medication	3 (3.2)	92
Analgesics	8 (8.7)	92
Vaccination		
Vaccinated status, *n* (%)	232 (82.9)	280
Vaccine type		
Spikevax® (Vaccine Moderna)	116 (50.0)	232
Comirnaty® (Vaccine Pfizer/Biotech)	114 (49.1)	232
COVID‐19 Vaccine Janssen® (Vaccine Johnson & Johnson)	2 (0.9)	232
Side effect vaccines		
Erythema injection site	124 (53.4)	232
Fever	78 (33.6)	232
Shivering	68 (29.3)	232
Skin alteration	38 (16.4)	232
Headache	121 (52.2)	232
Pain	122 (52.6)	232
Fatigue	114 (49.1)	232
Effect of vaccination on post‐COVID‐19 syndrome		
Improvement	70 (31.4)	223
Improvement in %, mean (95% CI)	42.9 (36.5–49.2)	70
Worsening	42 (18.8)	223
Worsening in %, mean (95% CI)	27.9 (23.3–32.5)	46
None	113 (50.7)	223
Reason for missing vaccination		
Fear of side effects	23 (47.9)	48
General fear	8 (16.7)	48
Fear of long‐term effects	23 (47.9)	48
Skepticism	8 (16.7)	48
Date not yet agreed	2 (4.2)	48

Abbreviations: 95% CI, 95% confidence interval; ESS, Epworth Sleepiness Scale; FSS, Fatigue Severity Score; PCR: polymerase chain reaction; WHO: World Health Organization.

^a^ *Source*: Education over 13 years corresponds to compulsory schooling (9 years) and job training (3 years) in Switzerland.

### 3.2 Phenotyping the three most frequent symptoms in respondents with post‐COVID‐19 syndrome

(I) Fatigue: The mean reported fatigue severity on FSS was 5.7 points (95% CI 5.5–5.9, range 0.7–7.0, *n* = 255). This was higher in female than in male respondents (mean, 95% CI, female 5.8, 5.6–6.0, *n* = 206; male 5.3, 5.0–5.7, *n* = 49, *p* = 0.003, Table S1). In most cases, participants experienced cognitive as well as motor fatigue. In 12.9% (32/249) and 5.2% (13/249) of cases, there was isolated motor or cognitive fatigue, respectively. Fatigue was more pronounced in the afternoon (morning 35/240, 14.6%; afternoon 100/240, 41.7%; evening 63/240, 26.3%; all day long 42/240, 17.5%). Participants with fatigue reported more frequently sleep disturbances than participants without fatigue (with fatigue: 148/244, (60.7%) vs. without fatigue 0/50, (0%), chi‐squared test, *p* < 0.001, Table S2). Participants with fatigue reported also more commonly pain (with fatigue: 184/247, (74.5%) vs. without fatigue 34/62, (54.8%), chi‐squared test, *p* = 0.005, Table S2) and were more often unable to work (with fatigue: 163/237, (68.8%) vs. without fatigue 6/31, (19.4%), chi‐squared test, *p* < 0.001, Table S2).

(II) Pain including headache: 6 months after the acute infection, 66.1% (193/292) of the participants reported pain. This decreased over time to 23.3% (45/193) being affected after 12‐month post‐COVID‐19. Of the participants who reported pain, 56/218 (25.7%) reported headache only and 53/218 (24.3%) reported musculoskeletal pain only, respectively. Patients reporting pain as a symptom of post‐COVID‐19 syndrome experienced more frequently pain during acute COVID‐19 illness than patients without pain in the context of post‐COVID‐19 syndrome. Participants with pain experienced more frequent and more severe fatigue (chi‐squared test & Mann–Whitney Test [MWT] each *p* < 0.001, Table S3). Participants with pain were also more frequently and during a longer duration incapacitated for work (Chi‐Quadrat test *p* < 0.001 and MWT *p* = 0.001, Table S3) compared with participants without pain.

(III) Sleep–wake disturbances: Sleep disturbances were reported by 50.3% of the participants (148/294). The mean Insomnia Severity Index (ISI) was 13.3 points (95% CI 12.4–14‐1, range 0–27.0, *n* = 241), and 91/241 (37.8%) were above the threshold for clinical insomnia (defined by ISI [17] ≥15 points). Excessive daytime sleepiness (defined by Epworth Sleepiness Scale (ESS) ≥11 points) was reported in 126/224 (56.3%) of the respondents. Participants with insomnia (defined by ISI ≥15 points) during post‐COVID‐19 syndrome compared with those without insomnia reported more frequently a disturbed sleep (with insomnia: 50/91, (54.9%) vs. without insomnia: 35/150 (23.3%), chi‐squared test, *p* < 0.001, Table S4). There was no association between insomnia and presence of fatigue (with insomnia: 91/91, (100.0%) vs. without insomnia: 148/150 (98.7%), chi‐squared test, *p* = 0.528, Table S4). However, if fatigue was reported, the fatigue intensity (measured by FSS) was more severe than in participants without insomnia (with insomnia: mean (95% CI) 6.1 (5.9–6.3), *n* = 91 vs. without insomnia mean (95% CI) 5.7 (5.5–5.9), *n* = 147, MWT *p* < 0.001, Table S4). No significant differences were present in respondents with and without excessive daytime sleepiness (Table S5).

### 3.3 Phenotyping mental disturbances and social aspects in respondents with post‐COVID‐19 syndrome

Almost one third (91/303, 30%) of respondents complained of depressed mood, and accordingly, 36.5% (113/309) of respondents consulted a psychiatrist or psychologist. Despite the generally high number of physicians consulted for post‐COVID‐19 syndrome (mean, (95% CI) 3.5, (3.2–3.8), range 0–25, *n* = 299), many respondents felt not taken seriously (106/258, 41.1%).

Post‐COVID‐19 syndrome also had an impact on work ability. More than half of the respondents (168/268, 62.7%) reported an inability to work, which lasted on average 26.6 weeks (95% CI 23.5–29.6, range 1–94, *n* = 168). Those people with reported inability to work also reported more commonly fatigue, sleep disturbances, and pain (Table S1–S4). Quality of life measured by WHO‐5 Well‐being Index was in overall low in respondents with post‐COVID‐19 syndrome (mean, (95% CI) 9.1, (8.5–9.8), range 1–25, *n* = 239, Table 1), and 175/239 (73.2%) of the respondents had scores below the WHO‐defined threshold for a reduced quality of life (cut‐off ≤13 points [18]).

### 3.4 Phenotyping therapeutic options for post‐COVID‐19 syndrome

**FIGURE 2:**
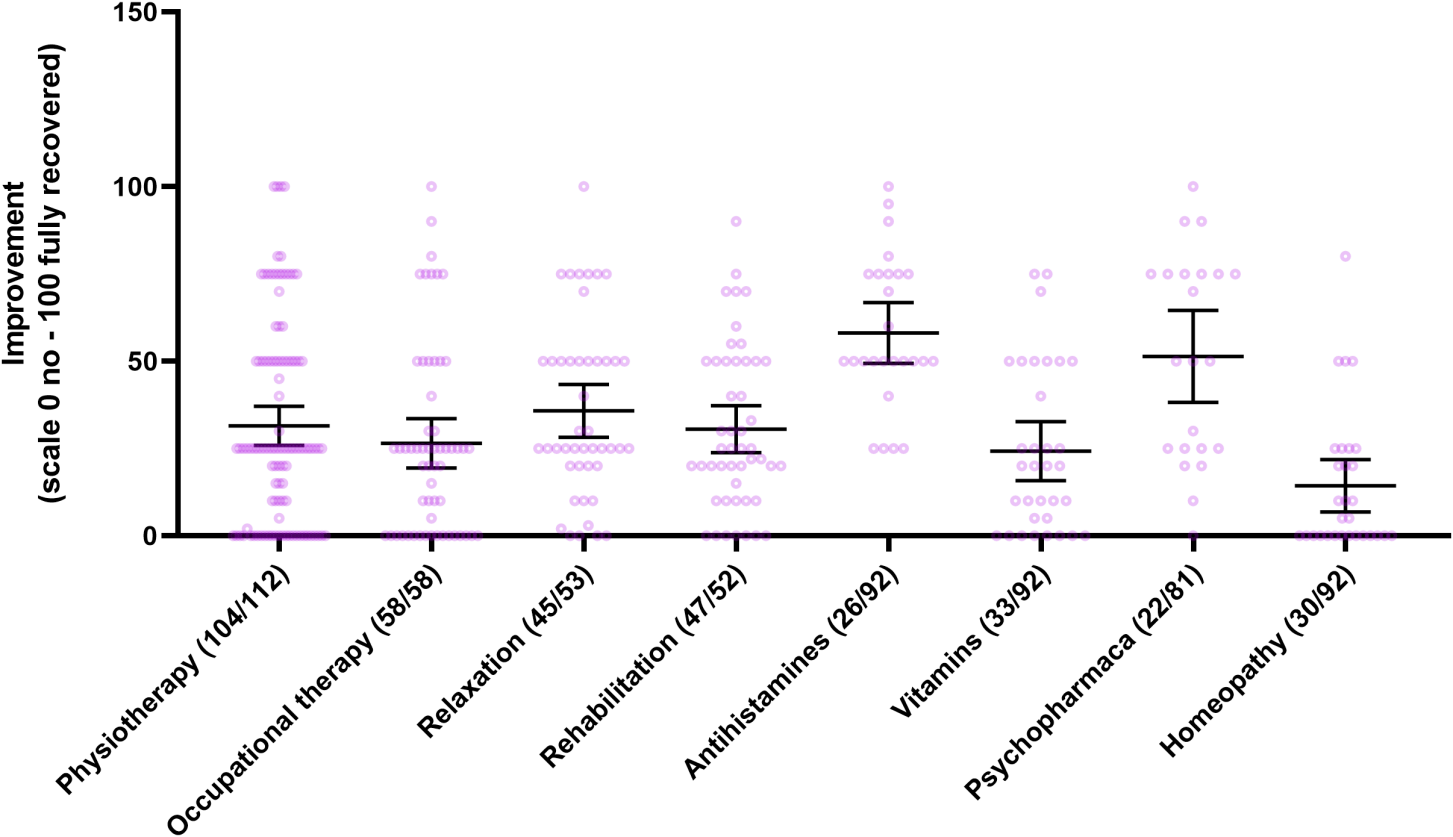
Effect of therapeutic interventions on post‐COVID‐19 symptoms

Of the non‐pharmacological measures, physiotherapy and relaxation techniques demonstrated efficacy on post‐COVID‐19 symptoms in our self‐reported questionnaire. The mean improvement in percentage was 31.5% (95% CI 25.9–37.1, range 0–100, *n* = 104) after physiotherapy and 35.8% (95% CI 28.2–43.4, range 0–100, *n* = 45) after relaxation techniques. Respondents rated their improvement due to occupational therapy as 26.5% (95% CI 19.4–33.6, range 0–100, *n* = 58) and rehabilitation as 30.6% (95% CI 23.8–37.3, range 0–90, *n* = 48, Table 1, Figure 2 and Table S6).

The most common medications reported were vitamins (35/92, 38.0%), antihistamines (27/92, 29.3%), inhalation medicines (23/92, 25.0%), and antidepressants (22/92, 23.9%, Table 1). The reported improvement in percentage were antihistamines 58.1% (95% CI, 49.4–66.8, range 25–100, *n* = 26), inhalation medicines 52.5% (95% CI, 42.4–62.6, range 10–80, *n* = 22), antidepressants 54.7% (95% CI, 41.6–67.8, range 0–100, *n* = 18), vitamins 24.2% (95% CI, 15.8–32.7, range 0–75, *n* = 33), and homeopathic formulations 14.3% (95% CI, 6.8–21.9, range 0–80, *n* = 30).

### 3.5 Phenotyping SARS‐CoV‐2 vaccination behavior in respondents with post‐COVID‐19 syndrome

**FIGURE 3:**
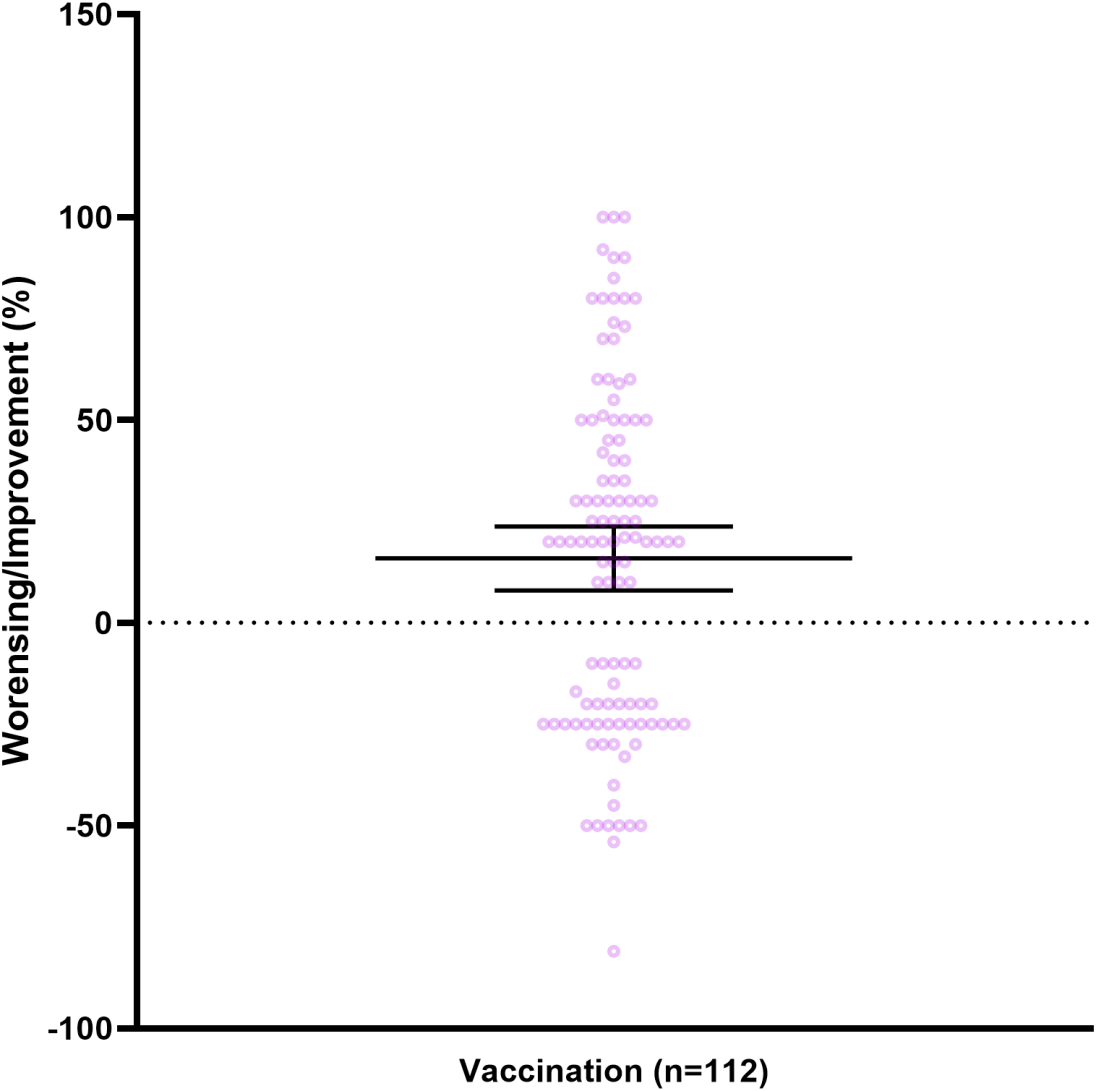
Effect of vaccination on post‐COVID‐19 symptoms

The majority of patients with post‐COVID‐19 syndrome received COVID‐19 vaccinations after post‐COVID‐19 diagnosis (232/280, 82.9%). In our study, male respondents were vaccinated significantly more often than female once (female: 117/219, (53.4%) vs. male: 49/51, (96.1%); *p* = 0.006, Table S1).No serious adverse reactions to vaccination were reported and the most common adverse reactions were injection site reaction (124/232, 53.4%), generalized pain (122/232, 52.6%), and headache (121/232, 52.2%). A slight majority of respondents reported no effect of vaccination on post‐COVID‐19 symptomatology (113/223, 50.7%). Seventy/223 respondents (31.4%) reported improvement in symptomatology after vaccination of on average 42.9% (95% CI 36.5–49.2, range 0–100, *n* = 70). Less than 20% (42/223, 18.8%) of respondents reported a worsening of symptoms after vaccination with a reported worsening of 27.9% (95% CI 23.3–32.5, range 0–81, *n* = 46). (Figure 3).

### 3.6 Phenotyping outcome and prognosis of the post‐COVID‐19 syndrome

**FIGURE 4:**
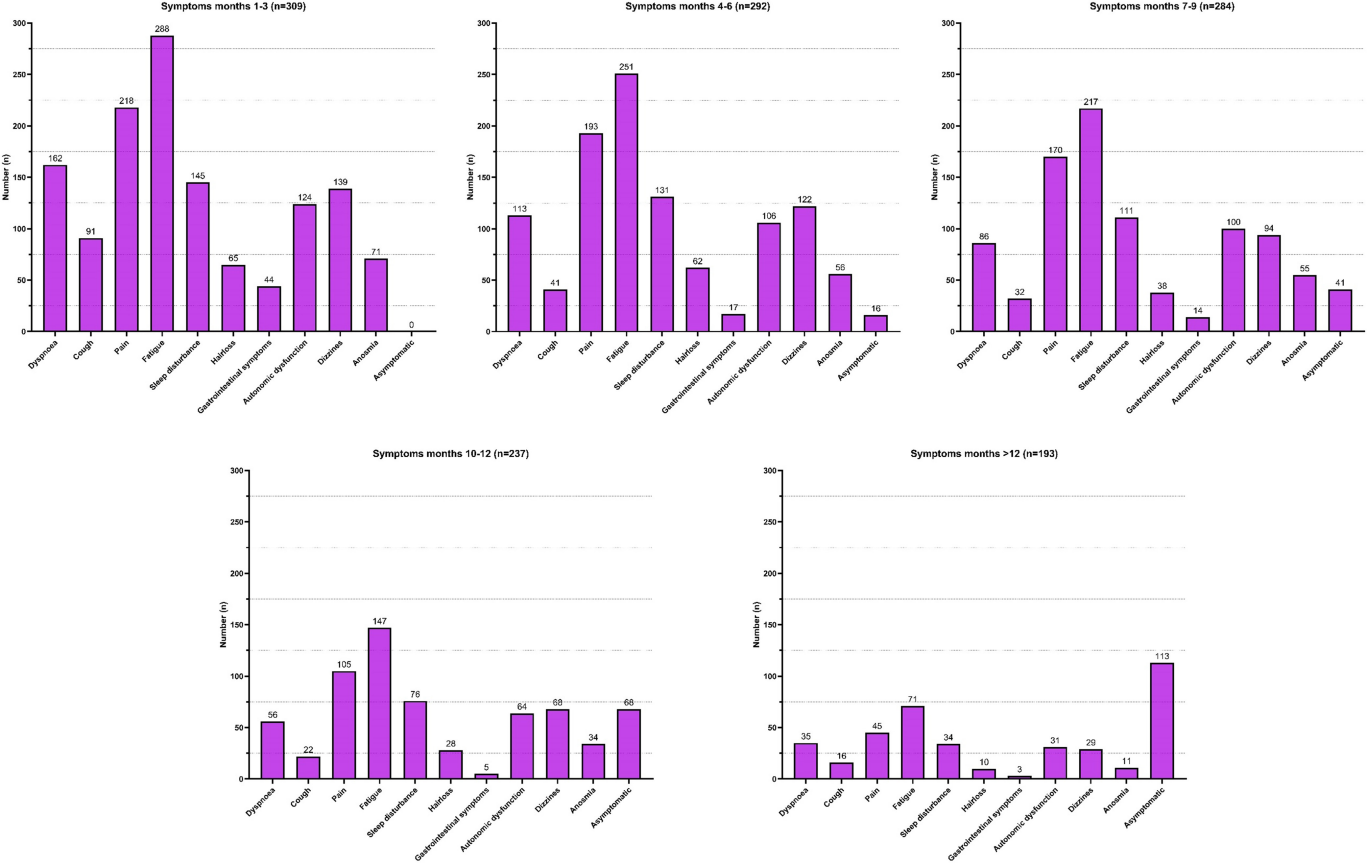
Development of post‐COVID‐19 symptoms over 12 months

The participants retrospectively rated their symptoms over a period of 12 months. We observed a decrease in the cumulative number of symptoms (time point 3 months: mean number of symptoms (95% CI) 4.7 (4.4–5.0), range 0–10, *n* = 309 vs. time point over 12 months mean (95% CI) 1.6 (1.3–1.9), range 0–9, *n* = 193). To note, over 12 months after COVID‐19, 58.5% (113/193) of respondents reported a complete disappearance of symptoms compared to 5.5% (16/292) after 4 months (chi‐squared test, *p* = 0.018) (Figure 4).

The majority (200/309, 64.7%) of the respondents reported an improvement (defined as a decrease in the cumulative number of symptoms reported of ≥2) between Month 3 and Month 12. Respondents without improvement were more likely not to have a laboratory‐proven SARS‐CoV‐2 infection by PCR or antigen testing (chi‐squared test, *p* = 0.004, Table S7). Stratifying our cohort in those with reported PCR or antigen test confirmed COVID‐19 syndrome and those where this confirmation was missing, we demonstrated a difference in the number of reported symptoms at 12 months. Here, respondents without laboratory‐confirmed infection had in total more remaining symptoms than those with proven infection (no confirmed infection vs. confirmed infection; mean (95% CI): 2.0 (1.3–2.6), range 0–7, *n* = 53 vs. 0.9 (0.7–1.2), and range 0–9, *n* = 256, MWT *p* < 0.001). Patients with laboratory‐confirmed infection reported more frequently comorbidities (no confirmed infection vs. confirmed infection 11/53, (20.7%) vs. 99/255, (38.8%), chi‐squared test, *p* = 0.008) and headache during acute COVID‐19 infection (no confirmed infection vs. confirmed infection: 20/53, (54.7%) vs. 201/256, (78.5%), chi‐squared test, *p* = 0.001). In addition, the time between acute COVID‐19 and completion of the questionnaire was longer in patients without confirmed infection. (MWT, *p* < 0.001). There were no differences in therapeutic strategies between these two groups (Table S8).

## 4 DISCUSSION

This study focuses on the characteristics and disease course in participants with post‐COVID‐19 syndrome in Switzerland based on an online survey of patient‐reported outcomes. This is the first Swiss study of its kind including all regions of Switzerland (Figure S1). The main findings are 1. Fatigue is the predominant symptom of post‐COVID‐19 syndrome, being present in >80% of respondents approximately 6 months after acute COVID‐19 infection; 2. More than 50% of the respondents reported a relevant reduction of symptoms after 12 months; 3. In the majority of patients, non‐pharmacological treatments recommended by NICE, such as physiotherapy, occupational therapy, relaxation methods, and rehabilitation showed positive effects on symptom relief.

Regarding fatigue, our observed prevalence as well as the improvement over time is well in line with previous studies reporting a range for presence of fatigue from 35 to 98%. [8, 9] Studies with higher reported fatigue frequencies such as Dennis et al. [10] performed the assessments closer to the acute COVID‐19 infection.

Association between time of observation and fatigue is also reflected in the decrease in fatigue in our work from 86.0% after 3 months to 36.8% after more than 12‐month post‐COVID‐19 diagnosis.

The mechanism how the SARS‐CoV‐2 virus leads to persistence of symptoms also after viral clearance causing the post‐COVID‐19 syndrome is still unclear. The current most plausible explanation is an inflammatory response as it was shown that mediators of inflammation in blood such as IFN‐β, PTX3, IFN‐γ, IFN‐λ2/3, and IL‐6 are associated with post‐COVID‐19 syndrome. [19]

When assessing fatigue in our cohort, the fatigue related to the non‐pharmaceutical interventions to control the spread of SARS‐CoV‐2 has to be considered as possible confounder [20, 21, 22] as they can also be associated with fatigue, which was recently recognized by the WHO and named Pandemic Fatigue. [23] However, we expect the impact on our survey to be rather low, as our survey lasted from October to December 2021 and the most social restriction measurements such as restrictions in social gathering stopped in Switzerland before the start of our survey on June 23, 2021. [24] However, post‐COVID‐19 fatigue also leads to a pronounced intolerance of performance and a corresponding reduction in the ability to work and activity in everyday life.

Fatigue often overlaps with sleep disturbances. In fact, in our study, 60.7% of patients with fatigue also reported sleep disturbances. In contrast, sleep disturbances were not reported frequently in patients without fatigue.

An interconnection between sleep disorders and fatigue is doubtless, in post‐COVID‐19 patients insomnia is most frequently observed. [20] Here, in agreement with previous data [21, 22, 23], our study also found that insomnia and excessive daytime sleepiness (EDS) are common symptoms of the post‐COVID‐19 syndrome affecting quality of life, highlighting the need for a structured post‐COVID‐19 consultation including sleep–wake questions.

We found a difference between respondents with and without laboratory proven post‐COVID‐19 syndrome. Respondents without relevant improvement of the symptoms over 1 year were more likely not to have laboratory‐proven SARS‐CoV‐2 infection. This highlights the need for a strict and thorough evaluation of other etiologies inpatients presenting with fatigue after COVID‐19 infection. We have also taken into account the initial low test capacity. This was significantly increased in the 12th week of 2020 (i.e., since 8th‐March‐2020). [24] In our cohort, only 12/309 (3.2%) were infected before this time. We therefore do not expect a relevant bias by the national testing capacities. Little is known about the risk factors for developing a post‐COVID‐19 syndrome. A recent study showed that bronchial asthma is a possible risk factor. [25] In our study, 11% of the participants had bronchial asthma. This percentage is higher than the prevalence of bronchial asthma in the Swiss population which ranges from 4.5% to 6.8%. [26] Thus, this could also be an indication that bronchial asthma might contribute to the development of post‐COVID‐19 syndrome.

Post‐COVID‐19 syndrome has a relevant effect on respondents ‘quality of life and ability to work. In our study, more than half of the respondents reported an inability to work, which persisted on average for almost 6 months. This is consistent with previous studies of non‐hospitalized patients with COVID‐19 reporting that about 12–23% remain absent from work at 3 and 7 months after acute COVID‐19. [7, 11] The inability to work lasts usually longer than 13 weeks [8, 10], which was also found in our work. In our survey, respondents with post‐COVID‐19 syndrome, reported also a relevant effect on the quality of life. Quality of life measured by WHO‐5 Well‐being Index was in overall low in respondents with post‐COVID‐19 syndrome and almost three quarters (73.2%) of the respondents reported scores below the WHO‐defined threshold for a reduced quality of life (cut‐off 13 points [18]). These results are in line with other studies highlighting the burden of the post‐COVID‐19 syndrome also for the society. [27, 28, 29] Regarding the treatment of the post‐COVID‐19 syndrome, the NICE recommends self‐management and support, including CBT and GET. [13] In our study, the non‐pharmacological measures (physiotherapy, relaxation techniques, occupational therapy, and rehabilitation) were reported to exert positive effects on post‐COVID‐19 symptoms supporting the NICE recommendations. Prospective studies focusing on treatment are urgently needed to confirm these findings.

Our study has limitations, which one has to bear in mind while interpreting the data. The online questionnaire was distributed through several different channels (e.g., Altea Long‐COVID Network, [14]), social media (e.g. Twitter and Facebook), and post‐COVID‐19 consultation at the (University Hospital Bern, Inselspital, Bern, Switzerland), which might have impacted our study population. The digital nature of the questionnaire, the distribution via self‐help groups, and social media might lead to a certain selection bias toward younger people with greater knowledge in the use of digital media, which should also be considered as limitation of our study. The response rate of our survey has to be highlighted as main limitation of our study. First as we openly distributed the survey via different channels, a response rate cannot be properly calculated as we do not know how many Swiss people were informed about our survey. Second considering the numbers of cases with post‐COVID‐19 syndrome, which can be estimated by percentage of COVID‐19 cases who develop a post‐COVID‐19 syndrome (28,406 to 63,411 [3]), we consider the response rate of our survey as low thus limiting the generalizability of our study. The main reason for referral to our outpatient consultation is prominent fatigue, which could also influence the selection of participants from this group. Furthermore, the self‐reported character of our survey has to be considered and has some limitation inherent to this type of study such as missing confirmation of post‐COVID‐19 diagnosis by a healthcare professional or validation of PCR or antigen tests, which were self‐reported by the survey respondents. [30] In addition, the mental effort to complete the survey (120 questions) may have also influenced the results, as severely affected people might have more frequently failed to complete the survey in comparison with less severely affected people. In this case, the severity and frequency of the reported symptoms in our study might even be underestimated. In sum, these results highlight the importance of further studies characterizing this syndrome as well as the need for prospective trials focusing on different treatment approaches. Evidence‐based recommendations are urgently needed to prepare the healthcare systems for the challenge of treating patients with post‐COVID‐19 syndrome.

## Patient and Public Involvement

It was not appropriate or possible to involve patients or the public in the design, or conduct, or reporting, or dissemination plans of our research.

## AUTHORS CONTRIBUTION

Diem Lara, MD, and Hoepner Robert, MD, PD, contributed to the design of the study, acquisition of data the analysis and interpretation of the data, and the writing and revision of the manuscript. Schwarzwald Anina, MD, Friedli Christoph, MD, Hammer Helly, MD, Gomes Fregolente Livia, MD, Warncke Jan, and Weber Lea contributed to the acquisition of data and revised the manuscript for intellectual content. Chan Andrew, MD, Prof., Bassetti Claudio, MD, Prof., and Salmen Anke, MD, PD, contributed to the interpretation of the data, and the writing and revision of the manuscript.

## CONFLICT OF INTERESTS

Diem Lara received travel grants from Merck, Biogen, Roche, and Bayer Switzerland. She also received speaker's honoraria from Biogen and Merck. Schwarzwald Anina, Gomes Fregolente Livia, Warncke Jan, and Weber Lea declare no conflict of interest related to this manuscript. Friedli Christoph received speaker honoraria and/or travel compensation for activities with Biogen, Sanofi Genzyme, Novartis, and Merck and research support from Chiesi, not related to this work. Hammer Helly received research support and travel grants from Biogen, Merck, Roche & BMS. Kamber Nicole received travel and/or speaker honoraria and served on advisory boards for Alexion, Biogen, Merck, Sanofi Genzyme, and Roche and received research support from Biogen. Chan Andrew received speakers'/board honoraria from Actelion (Janssen/J&J), Almirall, Bayer, Biogen, Celgene (BMS), Genzyme, Merck KGaA (Darmstadt, Germany), Novartis, Roche, and Teva, all for hospital research funds. He received research support from Biogen, Genzyme, and UCB, the European Union, and the Swiss National Foundation. He serves as associate editor of the European Journal of Neurology, on the editorial board for Clinical and Translational Neuroscience and as topic editor for the Journal of International Medical Research. Bassetti Claudio received speaker/advisor honoraria from JAZZ, Bioprojet, Takeda, Biogen, Rehaklinik Zihlschlacht and research support from the Swiss National Science Foundation (SNF) and the University of Bern. No conflicts of interest with this work. Salmen, Anke received speaker honoraria and/or travel compensation for activities with Bristol‐Myers Squibb, CSL Behring, Novartis, and Roche, and research support by the Baasch Medicus Foundation, the Medical Faculty of the University of Bern and the Swiss MS Society, not related to this work. Hoepner Robert received speaker/advisor honorary from Merck, Novartis, Roche, Biogen, Alexion, Sanofi, Janssen, Bristol‐Myers Squibb, and Almirall. He received research support within the last 5 years from Roche, Merck, Sanofi, Biogen, Chiesi, and Bristol‐Myers Squibb. He also received research grants from the Swiss MS Society and is a member of the Advisory Board of the Swiss MS Society. He also serves as associated editor for Journal of Central Nervous System disease. All conflicts are not related to this work.

## Supporting information


**Appendix S1** Supporting Information.

## Data Availability

Following an open data approach, anonymized data of the cohort can be requested via the corresponding author.
