# Supplementary material for: Multidimensional phenotyping of the post‐COVID‐19 syndrome: A Swiss survey study
Source: CNS Neurosci Ther. 2022 Aug 16;28(12):1953–63. doi: 10.1111/cns.13938 (PMC9538958; doi:10.1111/cns.13938)
Supplement: Supplementary file 1 — Appendix S1 Supporting Information. [file CNS-28-1953-s001.docx]

**Supplement Material**

| **Table of content** | **Page** |
| --- | --- |
| Supplement table 1: Comparison of female and male respondents in Post-COVID-19 syndrome. | 2 |
| Supplement table 2: Comparison of fatigued and not-fatigued respondents with Post-COVID-19 syndrome. | 5 |
| Supplement table 3: Comparison of respondents with and without pain in Post-COVID-19 syndrome | 6 |
| Supplement table 4: Comparison of respondents with and without insomnia (Insomnia Severity Index >15) in Post-COVID-19 syndrome. | 8 |
| Supplement table 5: Comparison of respondents with and without daytime sleepiness (ESS≥11) in Post-COVID-19 syndrome. | 10 |
| Supplement table 6: Effectiveness of therapies compared to homeopathy | 12 |
| Supplement table 7: Comparison of recovered and not-recovered respondents with Post-COVID-19 syndrome. | 13 |
| Supplement table 8: Comparison of respondents with and without confirmed infection Post-COVID-19 syndrome. | 15 |
| Supplement figure 1: Swiss canton’s distribution of respondent pro 100’000 inhabitants | 17 |

| **Variable** | **Female respondents** | **Male respondents** | **p-value** |
| --- | --- | --- | --- |
| **Patient characteristics** |  |  |  |
| Age, years, mean (95%CI), n | 44.6 (43.1-46.0), 249 | 45.1 (41.9-48.3), 60 | 0.874 |
| Comorbidities, n (%) | 97/249 (39.0) | 23/69 (33.3) | 1.0 |
| Positive PCR/Antigen Test, n (%) | 205/249 (82.3) | 51/60 (85.0) | 0.706 |
| **Symptoms of acute**  **COVID-19, n (%)** |  |  |  |
| Headache | 187/249 (75.1) | 43/60 (71.7) | 0.622 |
| Fever | 118/249 (47.4) | 34/60 (56.7) | 0.250 |
| Dyspnoea | 114/249 (45.8) | 19/60 (31.7) | 0.059 |
| Cough | 146/249 (58.6) | 32/60 (53.3) | 0.470 |
| Cold | 92/249 (36.9) | 14/60 (23.3) | 0.05 |
| Sore throat | 118/249 (47.4) | 26/60 (43.3) | 0.666 |
| Pain | 187/249 (75.1) | 35/60 (58.3) | 0.016 |
| Gastrointestinal symptoms | 66/249 (26.5) | 7/60 (1.0) | 0.017 |
| Fatigue | 226/249 (90.8) | 47/60 (78.3) | 0.012 |
| Sleep disturbance | 87/249 (34.9) | 16/60 (26.7) | 0.285 |
| Skin alteration | 24/249 (9.6) | 2/60 (3.3) | 0.191 |
| Anosmia | 74/249 (29.7) | 15/60 (25) | 0.069 |
| **Severity of COVID-19** |  |  |  |
| Hospitalisation, n (%) | 20/249 (8.0) | 13/60 (21.7) | **0.004** |
| Intubation, n (%) | 3/249 (1.2) | 3/60 (5.0) | 0.094 |
| Oxygen requirement, n (%) | 18/249 (7.2) | 11/60 (18.3) | 0.014 |
| ICU without intubation, n (%) | 88/249 (35.3) | 14/60 (23.3) | 0.025 |
| **Time variable** |  |  |  |
| Time between onset of acute infection and questionnaire, months, mean (95%CI), n | 13.1 (12.5-13.7), 242 | 12.9 (11.9-14.1), 57 | 0.798 |
| **Fatigue in Post-COVID-19 Syndrome** |  |  |  |
| Fatigue, n (%) | 198/246 (80.5) | 49/59 (83.1) | 0.716 |
| FSS, mean (95%CI), n | 5.8 (5.6-6.0), 206 | 5.3 (5.0-5.7), 49 | **0.003** |
| **Sleep disturbances and mental condition in Post-COVID-19 Syndrome** |  |  |  |
| Sleep disturbances, n (%) | 119/236 (50.4) | 29/58 (50.0) | 1.0 |
| Insomnia Severity Index, mean (95%CI), n | 13.6 (12.6-14.8), 193 | 12.0 (10.1-13.9), 48 | 0.072 |
| Sense of comprehension, n (%) | 84/214 (39.2) | 22/51 (49.0) | 0.636 |
| Incapacity to work, n (%) | 144/217 (66.4) | 25/50 (50.0) | 0.035 |
| Maximal incapacity to work, mean (95%CI) | 87.8 (83.8-91.9), 144 | 96.8 (91.6-102.0), 25 | 0.085 |
| Duration of incapacity to work, weeks, mean (95%CI) | 26.5 (23.2-29.9), 144 | 26.1 (18.0-34.2), 25 | 0.935 |
| **Pain in Post-COVID-19 Syndrome** |  |  |  |
| Pain, n (%) | 163/249 (65.4) | 36/60 (60.0) | 0.454 |
| **Therapies/Improvement** |  |  |  |
| Improvement physiotherapy in %, mean (95%CI) | 31.1 (25.3-36.8), 89 | 34.0 (13.3-54.8), 15 | 0.821 |
| Improvement occupational therapy in %, mean (95%CI), n | 27.1 (19.4-34.9), 52 | 20.8 (1.1-40.6), 6 | 0.835 |
| Improvement Relaxation methods in %, mean (95%CI) | 36.5 (28.7-44.3), 39 | 31.3 (-5.3-68.0), 6 | 0.456 |
| Improvement massage in %, mean (95%CI), n | 32.5 (24.3-40.7), 42 | 6.5 (-38.0-51.0), 2 | 0.178 |
| Improvement rehabilitation in %, mean (95%CI), n | 30.4 (23.2-37.7), 40 | 31.3 (8.6-53.9), 8 | 0.956 |
| Improvement antihistaminic medications in %, mean (95%CI), n | 55.2 (46.9-63.5), 24 | 92.5 (60.7-124.3) | 0.028 |
| **Vaccination** |  |  |  |
| Vaccination, n (%) | 117/219 (53.4) | 49/51 (96.1) | **0.006** |
| Improvement vaccination in %, mean (95%CI), n | 42.8 (35.4-50.1), | 47.9 (34.9-60.8), 14 | 0.345 |
| Worsening vaccination in %, mean (95%CI), n | 29.8 (24.0-35.6), 34 | 23.3 (16.5-30.1) | 0.686 |

*Supplement table 1: Comparison of female and male respondents in Post-COVID-19 syndrome.*

Abbreviations: 95%CI: 95% Confidence interval; FSS: Fatigue Severity Score; ICU: Intensive care unit; PCR: Polymerase chain reaction.

Statistic: Clinical phenomenology of fatigue and present comorbidities as well as serological findings were analysed using Mann-Whitney U Test (MWU) and Chi-Square-Test, respectively. Adjustment for multiple testing was performed by Bonferroni procedure in regard to each domain independently: patient characteristics: p-value < 0.016, acute infection: p-value < 0.004, severity of COVID-19 infection: p-value < 0.0125, time variable: p-value < 0.05, fatigue in Post-COVID-19 syndrome: p-value < 0.025, sleep disturbances and mental condition in Post-COVID-19 syndrome: p-value < 0.008; therapy: p-value < 0.007; vaccination: p-value < 0.017

| **Variable** | **Respondents with fatigue** | **Respondents without fatigue** | **p-value** |
| --- | --- | --- | --- |
| **Patient characteristics** |  |  |  |
| Age, years, mean (95%CI), n | 43.2 (43.1-46.1), 247 | 44.8 (41.8-47.7), 62 | 0.662 |
| Female sex, n (%) | 198/247 (80.2) | 51/62 (82.3) | 0.852 |
| Comorbidities, n (%) | 96/247 (38.9) | 14/62 (22.6) | 0.017 |
| Positive PCR/Antigen Test, n (%) | 203/247 (82.2) | 53/62 (85.5) | 0.706 |
| **Symptoms of acute**  **COVID-19, n (%)** |  |  |  |
| Headache | 185/247 (74.9) | 45/62 (72.6) | 0.745 |
| Fever | 127/247 (51.4) | 23/62 (37.1) | 0.155 |
| Dyspnoea | 108/247 (43.7) | 25/62 (40.3) | 0.669 |
| Cough | 148/247 (59.9) | 30/62 (48.4) | 0.706 |
| Cold | 89/247 (36.0) | 17/62 (27.4) | 0.233 |
| Sore throat | 123/247 (49.8) | 21/62 (33.9) | 0.032 |
| Pain | 182/247 (73.7) | 40/62 (64.5) | 0.158 |
| Gastrointestinal symptoms | 63/247 (25.5) | 10/62 (16.1) | 0.135 |
| Fatigue | 221/247 (89.5) | 52/62 (83.9) | 0.137 |
| Sleep disturbance | 87/247 (35.2) | 16/62 (25.8) | 0.177 |
| Skin alteration | 20/247 (8.1) | 6/62 (9.6) | 0.619 |
| Anosmia | 64/247 (25.9) | 24/62 (38.7) | 0.058 |
| **Severity of COVID-19** |  |  |  |
| Hospitalisation, n (%) | 27/247 (10.9) | 6/62 (9.7) | 1.0 |
| Intubation, n (%) | 3/247 (1.2) | 3/62 (4.8) | 0.102 |
| Oxygen requirement, n (%) | 23/247 (9.3) | 6/62 (9.7) | 1.0 |
| ICU without intubation, n (%) | 5/247 (2.0) | 3/62 (4.8) | 0.067 |
| **Time variable** |  |  |  |
| Time between onset of acute infection and questionnaire, months, mean (95%CI), n | 13.2 (12.6-13.7), 240 | 12.6 (11.7-13.6), 59 | 0.528 |
| **Sleep disturbances and mental condition in Post-COVID-19 Syndrome** |  |  |  |
| Sleep disturbances, n (%) | 148/244 (60.7) | 0/50 (0) | **<0.001°** |
| Insomnia Severity Index, mean (95%CI), n | 13.3 (12.5-14.1), 239 | 6.0 (-70.2-82.2), 2 | 0.795 |
| Sense of comprehension, n (%) | 95/235 (40.4) | 11/30 (36.7) | 0.844 |
| Incapacity to work, n (%) | 163/237 (68.8) | 6/31 (19.4) | **<0.001°°** |
| Maximal incapacity to work, mean (95%CI) | 89.7 (86.1-93.3), 163 | 75.0 (52.3-97.8), 6 | 0.017 |
| Duration of incapacity to work, weeks, mean (95%CI) | 27.0 (23.9-30.1,163 | 12.0 (1.7-22.3), 6 | 0.064 |
| **Pain in Post-COVID-19 Syndrome** |  |  |  |
| Pain, n (%) | 184/247 (74.5) | 34/62 (54.8) | **0.005** |
| **Therapies/Improvement** |  |  |  |
| Improvement physiotherapy in %, mean (95%CI) | 30.0 (24.0-36.0), 88 | 23.5 (13.2-33.8), 16 | 0.192 |
| Improvement occupational therapy in %, mean (95%CI), n | 25.6 (17.7-33.5), 50 | 31.9 (14.9-48.9), 8 | 0.333 |
| Improvement Relaxation methods in %, mean (95%CI) | 35.3 (27.0-43.6), 39 | 38.8 (12.7-65.0), 6 | 0.520 |
| Improvement massage in %, mean (95%CI), n | 30.9 (21.9-39.8), 35 | 33.1 (11.3-55.0), 9 | 0.723 |
| Improvement rehabilitation in %, mean (95%CI), n | 30.9 (21.9-39.8), 35 | 33.1 (11.3-55.0), 9 | 0.136 |
| Improvement antihistaminic medications in %, mean (95%CI), n | 60.7 (51.6-69.8), 22 | 43.7 (5.7- 81.8), 4 | 0.174 |
| Decrease of Post-COVID-19 symptoms (≥2), n (%) | 156/247 (63.2) | 42/62 (67.7) | 0.656 |
| **Vaccination** |  |  |  |
| Vaccination, n (%) | 202/238 (84.9) | 24/32 (75.0) | 0.199 |
| Improvement vaccination in %, mean (95%CI), n | 42.8 (36.3-49.1), 67 | 44.3 (-34.1-122.7), 3 | 0.930 |
| Worsening vaccination in %, mean (95%CI), n | 27.1 (22.1-32.1), 40 | 33.0 (17.3-48.7), 6 | 0.353 |

*Supplement table 2: Comparison of fatigued and not-fatigued* respondents *with Post-COVID-19 syndrome.*

Abbreviations: 95%CI: 95% Confidence interval; ESS: Epworth Sleepiness Scale; FSS: Fatigue Severity Score; ICU: Intensive care unit; PCR: Polymerase chain reaction..

Statistic: Clinical phenomenology of fatigue and present comorbidities as well as serological findings were analysed using Mann-Whitney U Test (MWU) and Chi-Square-Test, respectively. Adjustment for multiple testing was performed by Bonferroni procedure in regard to each domain independently: patient characteristics: p-value < 0.0125, acute infection: p-value < 0.004, severity of COVID-19 infection: p-value < 0.0125, time variable: p-value < 0.05, sleep disturbances and mental condition in Post-COVID-19 syndrome: p-value < 0.008; pain in Post-COVID-19 syndrome: p-value < 0.05; therapy: p-value < 0.007; vaccination: p-value < 0.017. Exact p-values given: **°**3.7752E-18; **°°**0.000001.

| **Variable** | **Respondents with pain** | **Respondents without pain** | **p-value** |
| --- | --- | --- | --- |
| **Patients characteristics** |  |  |  |
| Age, years, mean (95%CI), n | 45.0 (43.4-46.6), 218 | 44.0 (41.6-46.3), 91 | 0.485 |
| Female sex, n (%) | 182/218 (83.5) | 67/91 (73.6) | 0.058 |
| Comorbidities, n (%) | 87/218 (39.9) | 23/91 (25.3) | 0.014 |
| Positive PCR/Antigen Test, n (%) | 184/218 (84.4) | 72/91 (79.1) | 0.320 |
| **Symptoms of acute COVID-19, n (%)** |  |  |  |
| Headache | 178/218 (81.6) | 52/91 (57.1) | **<0.001°** |
| Fever | 112/218 (51.4) | 40/91 (44.0) | 0.262 |
| Dyspnoea | 101/218 (46.3) | 32/91 (35.2) | 0.078 |
| Cough | 126/218 (57.8) | 52/91 (57.1) | 1.0 |
| Cold | 73/218 (29.4) | 33/91 (36.3) | 0.694 |
| Sore throat | 107/218 (49.1) | 37/91 (40.7) | 0.211 |
| Pain | 174/218 (79.8) | 48/91 (52.7) | **<0.001°°** |
| Gastrointestinal symptoms | 61/218 (28.0) | 12/91 (13.2) | 0.005 |
| Fatigue | 195/218 (89.4) | 78/91 (85.7) | 0.339 |
| Sleep disturbance | 85/218 (39.0) | 18/91 (19.8) | **0.001** |
| Skin alteration | 23/218 (10.6) | 3/91 (3.2) | 0.042 |
| Anosmia | 59/218 (27.1) | 29/91 (31.8) | 0.692 |
| **Severity of COVID-19** |  |  |  |
| Hospitalisation, n (%) | 28/218 (12.8) | 5/91 (5.5) | 0.068 |
| Intubation, n (%) | 2/218 (0.9) | 4/91 (4.4) | 0.120 |
| Oxygen requirement, n (%) | 24/218 (11.0) | 5/91 (5.5) | 0.197 |
| ICU without intubation, n (%) | 4/218 (1.8) | 4/91 (4.4) | 0.334 |
| **Time variable** |  |  |  |
| Time between onset of acute infection and questionnaire, months, mean (95%CI), n | 13.4 (12.7-14.2), 145 | 12.6 (11.9-13.3), 139 | 0.669 |
| **Fatigue in Post-COVID-19 Syndrome** |  |  |  |
| Fatigue, n (%) | 184/218 (84.4) | 63/91 (69.2) | **0.005** |
| FSS, mean (95%CI), n | 5.9 (5.8-6.1), 188 | 5.1 (4.7-5.5), 67 | **<0.001°°°** |
| **Sleep disturbances and mental condition in Post-COVID-19 Syndrome** |  |  |  |
| Sleep disturbances, n (%) | 114/212 (53.8) | 34/82 (41.5) | 0.069 |
| Insomnia Severity Index, mean (95%CI), n | 13.7 (12.7-14.7), 181 | 11.9 (10.3-13.5), 60 | 0.076 |
| Sense of comprehension, n (%) | 74/190 (38.9) | 32/75 (42.7) | 0.581 |
| Incapacity to work, n (%) | 130/191 (68.1) | 39/76 (51.3) | **0.008** |
| Maximal incapacity to work, mean (95%CI) | 90.7 (86.7-94.6), 130 | 84.1 (75.9-92.3), 47 | 0.086 |
| Duration of incapacity to work, weeks, mean (95%CI) | 28.9 (25.4-32.3), 130 | 18.3 (12.4-24.3), 39 | **0.001** |
| **Therapies** |  |  |  |
| Improvement physiotherapy in %, mean (95%CI) | 28.0 (21.2-34.8), 67 | 35.6 (20.6-51.1), 17 | 0.494 |
| Improvement occupational therapy in %, mean (95%CI), n | 25.6 (17.7-33.6), 49 | 31.1 (13.3-48.9), 9 | 0.368 |
| Improvement Relaxation methods in %, mean (95%CI) | 37.6 (29.5-36.9), 39 | 23.8 (-4.5-52.2),6 | 0.131 |
| Improvement massage in %, mean (95%CI), n | 34.4 (25.0-43.7), 32 | 23.2 (6.2-40.1), 12 | 0.152 |
| Improvement rehabilitation in %, mean (95%CI), n | 31.7 (24.7-38.7), 40 | 25.0 (0.12-49.9), 8 | 0.296 |
| Improvement antihistaminic medications in %, mean (95%CI), n | 61.0 (51.8-70.2), 20 | 48.3 (20.6- 76.0), 6 | 0.361 |
| Improvement over the time, n (%) | 142/218 (65.1) | 58/91 (63.7) | 0.896 |
| **Vaccination** |  |  |  |
| Vaccination, n (%) | 165/194 (85.1) | 61/76 (80.3) | 0.362 |
| Improvement vaccination in %, mean (95%CI), n | 40.5 (33.9-47.1), 58 | 54.3 (34.1-74.6), 12 | 0.126 |
| Worsening vaccination in %, mean (95%CI), n | 28.3 (22.5-34.0), 36 | 26.5 (19.8-33.3), 10 | 0.892 |

*Supplement table 3: Comparison of respondents with and without pain in Post-COVID-19 syndrome.*

Abbreviations: 95%CI: 95% Confidence interval; FSS: Fatigue Severity Score; ICU: Intensive care unit; PCR: Polymerase chain reaction.

Statistic: Clinical phenomenology of fatigue and present comorbidities as well as serological findings were analysed using Mann-Whitney U Test (MWU) and Chi-Square-Test, respectively. Adjustment for multiple testing was performed by Bonferroni procedure in regard to each domain independently: patient characteristics: p-value < 0.0125, acute infection: p-value < 0.004, severity of COVID-19 infection: p-value < 0.0125, time variable: p-value < 0.05, fatigue in Post-COVID-19 syndrome: p-value < 0.025, sleep disturbances and mental condition in Post-COVID-19 syndrome: p-value < 0.008; therapy: p-value < 0.007; vaccination: p-value < 0.017

Exact p-values given: **°**0.000014; **°°**0.000003; **°°°**0.000016;

| **Variable** | **Respondents with insomnia** | **Respondents without insomnia** | **p-value** |
| --- | --- | --- | --- |
| **Patients characteristics** |  |  |  |
| Age, years, mean (95%CI), n | 45.2 (42.8-47.6), 91 | 44.6 (42.6-46.7), 150 | 0.621 |
| Female sex, n (%) | 79/91 (86.8) | 114/150 (76.0) | 0.047 |
| Comorbidities, n (%) | 38/91 (41.8) | 56/150 (37.3) | 0.586 |
| Positive PCR/Antigen Test, n (%) | 76/91 (83.5) | 122/150 (81.3) | 0.731 |
| **Symptoms of acute COVID-19, n (%)** |  |  |  |
| Headache | 73/91 (80.2) | 106/150 (70.7) | 0.128 |
| Fever | 48/91 (52.7) | 75/150 (50.0) | 0.692 |
| Dyspnoea | 46/91 (50.5) | 59/150 (39.3) | 0.108 |
| Cough | 58/91 (63.7) | 85/150 (56.7) | 0.344 |
| Cold | 37/91 (40.7) | 47/150 (31.3) | 0.164 |
| Sore throat | 51/91 (56.0) | 68/150 (45.3) | 0.113 |
| Pain | 70/91 (76.9) | 107/150 (71.3) | 0.370 |
| Gastrointestinal symptoms | 31/91 (34.1) | 31/150 (20.7) | 0.134 |
| Fatigue | 85/91 (93.4) | 130/150 (86.7) | 0.134 |
| Sleep disturbance | 50/91 (54.9) | 35/150 (23.3) | **<0.001°** |
| Skin alteration | 13/91 (14.3) | 6/150 (4.0) | 0.006 |
| Anosmia | 22/91 (24.2) | 39/150 (26.0) | 0.528 |
| **Severity of COVID-19** |  |  |  |
| Hospitalisation, n (%) | 7/91 (7.7) | 19/150 (12.7) | 0.286 |
| Intubation, n (%) | 8/90 (8.9) | 15/149 (10.1) | 0.825 |
| Oxygen requirement, n (%) | 1/89 (1.1) | 2/148 (1.4) | 1.0 |
| ICU without intubation, n (%) | 1/89 (1.1) | 4/148 (2.7 | 0.526 |
| **Time variable** |  |  |  |
| Time between onset of acute infection and questionnaire, months, mean (95%CI), n | 13.7 (12.7-14.7), 91 | 12.8 (12.1-13.5), 150 | 0.197 |
| **Fatigue in Post-COVID-19 Syndrome** |  |  |  |
| Fatigue, n (%) | 91/91 (100.0) | 148/150 (98.7) | 0.528 |
| FSS, mean (95%CI), n | 6.1 (5.9-6.3), 91 | 5.7 (5.5-5.9), 147 | **<0.001°°** |
| **Mental condition in Post-COVID-19 Syndrome** |  |  |  |
| Sense of comprehension, n (%) | 31/89 (34.8) | 65/146 (44.5) | 0.172 |
| Incapacity to work, n (%) | 69/89 (77.5) | 93/147 (63.2) | 0.030 |
| Maximal incapacity to work, mean (95%CI) | 89.5 (83.5-95.5), 69 | 90.3 (85.9-94.7), 93 | 0.910 |
| Duration of incapacity to work, weeks, mean (95%CI), n | 29.7 (24.3-35.1), 69 | 25.2 (21.5-30.0), 93 | 0.386 |
| **Pain in Post-COVID-19 Syndrome** |  |  |  |
| Pain, n (%) | 62/91 (68.1) | 77/150 (51.3) | **0.011** |
| **Therapies** |  |  |  |
| Improvement physiotherapy in %, mean (95%CI) | 25.3 (15.6-35.5), 35 | 33.0 (25.3-40.8), 53 | 0.172 |
| Improvement occupational therapy in %, mean (95%CI), n | 20.9 (13.0-38.8), 22 | 28.0 (16.9-39.2), 28 | 0.756 |
| Improvement Relaxation methods in %, mean (95%CI) | 34.2 (21.0-47.4), 16 | 35.0 (29.3-46.1) | 0.877 |
| Improvement massage in %, mean (95%CI), n | 30.3 (18.1-42.5), 16 | 28.9 (17.1-40.7), 18 | 0.905 |
| Improvement rehabilitation in %, mean (95%CI), n | 29.4 (17.7-41.2), 18 | 30.7 (21.2-38.9), 28 | 0.717 |
| Improvement antihistaminic medications in %, mean (95%CI), n | 61.9 (50.3-73.5), 13 | 58.9 (40.9-76.9), 9 | 0.972 |
| Improvement over the time, n (%) | 54/91 (59.3) | 100/150 (66.7) | 0.270 |
| **Vaccination** |  |  |  |
| Vaccination, n (%) | 80/90 (88.9) | 122/148 (82.4) | 0.197 |
| Improvement vaccination in %, mean (95%CI), n | 45.0 (34.5-55.5), 32 | 40.9 (32.5-49.2), 35 | 0.806 |
| Worsening vaccination in %, mean (95%CI), n | 29.3 (19.8-38.9), 16 | 25.7 (19.6-31.7), 24 | 0.388 |

*Supplement table 4: Comparison of respondents with and without insomnia (Insomnia Severity Index >15) in Post-COVID-19 syndrome.*

Abbreviations: 95%CI: 95% Confidence interval;; FSS: Fatigue Severity Score; ICU: Intensive care unit; PCR: Polymerase chain reaction;.

Statistic: Clinical phenomenology of fatigue and present comorbidities as well as serological findings were analysed using Mann-Whitney U Test (MWU) and Chi-Square-Test, respectively. Adjustment for multiple testing was performed by Bonferroni procedure in regard to each domain independently: patient characteristics: p-value < 0.0125, acute infection: p-value < 0.004, severity of COVID-19 infection: p-value < 0.0125, time variable: p-value < 0.05, fatigue in Post-COVID-19 syndrome: p-value < 0.025, mental condition in Post-COVID-19 syndrome: p-value < 0.02; pain in Post-COVID-19 syndrome: p-value < 0.05; therapy: p-value < 0.007; vaccination: p-value < 0.017

Exact p-values given: **°** 9.1984E-7; **°°** 0.000109

| **Variable** | **Respondents with excessive daytime sleepiness (ESS≥11)** | **Respondents without excessive daytime sleepiness (ESS<11)** | **p-value** |
| --- | --- | --- | --- |
| **Patients characteristics** |  |  |  |
| Age, years, mean (95%CI), n | 43.5 (41.3-45.7), 126 | 47.0 (44.7-49.3), 98 | 0.050 |
| Female sex, n (%) | 102/126 (81.0) | 81/98 (82.7) | 0.862 |
| Comorbidities, n (%) | 54/126 (42.9) | 37/98 (37.8) | 0.495 |
| Positive PCR/Antigen Test, n (%) | 111/126 (88.1) | 78/98 (79.6) | 0.096 |
| **Symptoms of acute COVID-19, n (%)** |  |  |  |
| Headache | 97/126 (77.0) | 69/98 (70.4) | 0.285 |
| Fever | 79/126 (62.7) | 35/98 (35.7) | **0.001** |
| Dyspnoea | 60/126 (47.6) | 43/98 (43.9) | 0.592 |
| Cough | 80/126 (63.5) | 56/98 (57.1) | 0.339 |
| Cold | 46/126 (36.5) | 35/98 (35.7) | 1.0 |
| Sore throat | 62/126 (49.2) | 48/98 (49.0) | 1.0 |
| Pain | 95/126 (75.4) | 69/98 (70.4) | 0.448 |
| Gastrointestinal symptoms | 29/126 (23.0) | 29/98 (29.6) | 0.285 |
| Fatigue | 113/126 (89.7) | 86/98 (87.8) | 0.674 |
| Sleep disturbance | 46/126 (36.5) | 38/98 (38.8) | 0.781 |
| Skin alteration | 9/126 (7.1) | 10/98 (10.2) | 0.473 |
| Anosmia | 35/126 (27.8) | 15/98 (15.3) | 0.399 |
| **Severity of COVID-19** |  |  |  |
| Hospitalisation, n (%) | 14/126 (11.1) | 12/98 (12.2) | 0.835 |
| Intubation, n (%) | 1/126 (0.8) | 2/98 (2.0) | 0.580 |
| Oxygen requirement, n (%) | 14/126 (11.1) | 9/98 (9.2) | 0.665 |
| ICU without intubation, n (%) | 3/126 (2.4) | 2/98 (2.0) | 0.624 |
| **Time variable** |  |  |  |
| Time between onset of acute infection and questionnaire, months, mean (95%CI), n | 12.8 (12.0-13.6), 126 | 13.2 (12.4-14.0), 98 | 0.816 |
| **Fatigue in Post-COVID-19 Syndrome** |  |  |  |
| Fatigue, n (%) | 126/126 (100.0) | 97/98 (99.0) | 0.437 |
| FSS, mean (95%CI), n | 5.9 (5.8-6.1), 126 | 5.7 (5.45.9), 96 | 0.175 |
| **Mental condition in Post-COVID-19 Syndrome** |  |  |  |
| Sense of comprehension, n (%) | 51/126 (40.5) | 38/94 (40.4) | 1.0 |
| Incapacity to work, n (%) | 91/125 (72.8) | 62/96 (64.6) | 0.122 |
| Maximal incapacity to work, mean (95%CI) | 91.5 (87.2-95.8), 91 | 88.7 (82.6-95.0), 63 | 0.448 |
| Duration of incapacity to work, weeks, mean (95%CI), n | 26.3 (22.4-30.1), 91 | 27.9 (22.6-33.4), 62 | 0.753 |
| **Pain in Post-COVID-19 Syndrome** |  |  |  |
| Pain, n (%) | 98/126 (77.8) | 75/98 (76.5) | 0.873 |
| **Therapies** |  |  |  |
| Improvement physiotherapy in %, mean (95%CI) | 28.8 (19.6-38.1), 43 | 31.2 (22.7-39.8), 38 | 0.399 |
| Improvement occupational therapy in %, mean (95%CI), n | 17.6 (10.3-24.9), 23 | 35.2 (20.2-50.2), 22 | 0.120 |
| Improvement Relaxation methods in %, mean (95%CI) | 34.3 (21.6-46.9), 20 | 34.3 (22.5-46.1) | 0.919 |
| Improvement massage in %, mean (95%CI), n | 33.0 (17.7-48.3), 15 | 27.1 (16.7-37.4), 17 | 0.602 |
| Improvement rehabilitation in %, mean (95%CI), n | 25.4 (15.9-35.3), 21 | 35.1 (24.7-45.6), 23 | 0.241 |
| Improvement antihistaminic medications in %, mean (95%CI), n | 66.2 (54.6-77.7), 13 | 57.1 (37.9-76.4), 7 | 0.485 |
| Improvement over the time, n (%) | 75/126 (59.5) | 70/98 (71.4) | 0.069 |
| **Vaccination** |  |  |  |
| Vaccination, n (%) | 102/126 (81.0) | 85/98 (86.7) | 0.361 |
| Improvement vaccination in %, mean (95%CI), n | 41.1 (30.9-51.3), 31 | 43.6 (24.2-53.1), 32 | 0.710 |
| Worsening vaccination in %, mean (95%CI), n | 28.9 (19.2-38.5), 17 | 26.7 (20.2-33.2), 20 | 0.869 |

*Supplement table 5: Comparison of respondents with and without daytime sleepiness (ESS≥11) in Post-COVID-19 syndrome.*

Abbreviations: 95%CI: 95% Confidence interval; ESS: Epworth Sleepiness Scale; FSS: Fatigue Severity Score; ICU: Intensive care unit; PCR: Polymerase chain reaction.

Statistic: Clinical phenomenology of fatigue and present comorbidities as well as serological findings were analysed using Mann-Whitney U Test (MWU) and Chi-Square-Test, respectively. Adjustment for multiple testing was performed by Bonferroni procedure in regard to each domain independently: patient characteristics: p-value < 0.0125, acute infection: p-value < 0.004, severity of COVID-19 infection: p-value < 0.0125, time variable: p-value < 0.05, fatigue in Post-COVID-19 syndrome: p-value < 0.025, mental condition in Post-COVID-19 syndrome: p-value < 0.05; pain in Post-COVID-19 syndrome: p-value < 0.05; therapy: p-value < 0.007; vaccination: p-value < 0.017

| **Improvement homeopathy in %, mean (95%CI), n** | **Improvement** **physiotherapy in %, mean (95%CI)** |  |
| --- | --- | --- |
| 14.3 (6.8-21.9), 30 | 31.5 (25.9-37.1), 104 | **0.009** |
|  | **Improvement occupational therapy in %, mean (95%CI)** |  |
|  | 35.8 (28.2-43.4), 58 | 0.197 |
|  | **Improvement relaxation methods in %, mean (95%CI), n** |  |
|  | 35.8 (28.2-43.4), 45 | **0.001** |
|  | **Improvement rehabilitation in %, mean (95%CI), n** |  |
|  | 30.6 (23.8-37.3), 47 | 0.0225 |
|  | **Improvement antihistaminic medications in %, mean (95%CI), n** |  |
|  | 58.1 (49.4-66.8), 26 | **<0.001°** |
|  | **Improvement vitamins in %, mean (95%CI), n** |  |
|  | 24.2 (15.8-32.7), 33 | 0.724 |
|  | **Improvement antidepressants in %, mean (95%CI), n** |  |
|  | 54.7 (41.6-67.8), 22 | **<0.001°°** |

*Supplement table 6: Effectiveness of therapies compared to homeopathy*

Abbreviations: 95%CI: 95% Confidence interval

Statistic: Kruskal-Wallis-Test

Exact p-values given: **°**0.0001; **°°** 0.0001

| **Variable** | **Respondents with Improvement** | **Respondents without improvement** | **p-value** |
| --- | --- | --- | --- |
| **Patient Characteristics** |  |  |  |
| Age, years, mean (95%CI), n | 44.9 (43.2-46.6), 200 | 44.3 (42.1-46.4), 109 | 0.645 |
| Female sex, n (%) | 163/200 (81.5) | 86/109 (78.9) | 0.652 |
| Comorbidities, n (%) | 75/200 (37.5) | 35/109 (32.1) | 0.384 |
| Positive PCR/Antigen Test, n (%) | 175/200 (87.5) | 81/109 (74.3) | **0.004** |
| **Symptoms of acute COVID-19, n (%)** |  |  |  |
| Headache | 150/200 (75.0) | 80/109 (73.4) | 0.786 |
| Fever | 93/200 (46.5) | 59/109 (54.1) | 0.234 |
| Dyspnoea | 76/200 (38.0) | 57/109 (52.3) | 0.017 |
| Cough | 110/200 (55.0) | 68/109 (62.4) | 0.230 |
| Cold | 69/200 (34.5) | 37/109 (33.9) | 0.922 |
| Sore throat | 94/200 (47.0) | 50/109 (45.9) | 0.905 |
| Pain | 144/200 (72.0) | 78/109 (71.6) | 0.934 |
| Gastrointestinal symptoms | 42/200 (21.0) | 31/109 (28.4) | 0.162 |
| Fatigue | 181/200 (90.5) | 92/109 (84.4) | 0.137 |
| Sleep disturbance | 63/200 (31.5) | 40/109 (36.7) | 0.378 |
| Skin alteration | 15/200 (7.5) | 11/109 (10.1) | 0.521 |
| Anosmia | 61/200 (30.5) | 27/109 (24.8) | 0.295 |
| **Severity of COVID-19** |  |  |  |
| Hospitalisation, n (%) | 23/200 (11.5) | 10/109 (9.1) | 0.570 |
| Intubation, n (%) | 5/200 (2.5) | 1/109 (0.9) | 0.426 |
| Oxygen requirement, n (%) | 19/200 (9.5) | 10/109 (9.1) | 0.934 |
| ICU without intubation, n (%) | 6/200 (3.0) | 2/109 (1.8) | 0.637 |
| **Time variable** |  |  |  |
| Time between onset of acute infection and questionnaire, months, mean (95%CI), n | 12.6 (12.1-13.0), 200 | 14.1 (12.9-15.2), 109 | **0.006** |
| **Fatigue in Post-COVID-19 Syndrome** |  |  |  |
| Fatigue, n (%) | 158/200 (79.0) | 89/109 (81.7) | 0.542 |
| FSS, mean (95%CI), n | 5.6 (5.4-5.8), 163 | 5.9 (5.6-6.1), 92 | 0.056 |
| **Sleep disturbances and mental condition in Post-COVID-19 Syndrome** |  |  |  |
| Sleep disturbances, n (%) | 92/200 (46.0) | 56/109 (51.4) | 0.467 |
| Insomnia Severity Index, mean (95%CI), n | 12.9 (11.9-14.0), 154 | 13.9 (12.5-15.2), 87 | 0.289 |
| ESS, mean (95%CI), n | 10.5 (9.8-11.3), 145 | 11.6 (10.6-12.7), 79 | 0.077 |
| WHO-5 Well-being Index, mean (95%CI), n | 9.7 (8.8-10.5), 154 | 8.1 (7.1-9.1), 85 | 0.018 |
| Sense of comprehension, n (%) | 77/200 (38.5) | 29/109 (26.6) | 0.048 |
| **Therapy** |  |  |  |
| Improvement physiotherapy in %, mean (95%CI) | 35.7 (29.1-42.3), 68 | 23.5 (13.2-33.8), 36 | 0.011 |
| Improvement occupational therapy in %, mean (95%CI), n | 26.7 (17.6-35.9), 38 | 26.0 (13.9-38.1), 20 | 1.0 |
| Improvement Relaxation methods in %, mean (95%CI) | 42.8 (32.9-52.8), 28 | 24.1 (13.6-34.7), 17 | 0.016 |
| Improvement massage in %, mean (95%CI), n | 33.5 (23.3-46.3), 29 | 27.2 (12.9-41.5), 15 | 0.571 |
| Improvement rehabilitation in %, mean (95%CI), n | 30.9 (21.6-38.6), 33 | 31.6 (19.5-43.7), 15 | 0.622 |
| Improvement antihistaminic medications in %, mean (95%CI), n | 56.7 (46.2-67.3), 17 | 60.6 (41.8- 79.3), 17 | 0.833 |
| Improvement inhalation medicine in %, mean (95%CI), n | 55.9 (40.5-71.3), 11 | 49.1 (33.5-64.7), 11 | 0.562 |
| Improvement antidepressants in %, mean (95%CI), n | 52.3 (36.9-67.8), 17 | 48.0 (8.1-81.9), 5 | 0.905 |
| **Vaccination** |  |  |  |
| Vaccination, n (%) | 154/177 (87.0) | 72/93 (77.4) | 0.034 |
| Improvement vaccination in %, mean (95%CI), n | 44.6 (37.5-51.7), 50 | 38.6 (24.5-52.7), 20 | 0.169 |
| Worsening vaccination in %, mean (95%CI), n | 27.1 (2.1-33.1), 26 | 28.9 (21.1-36.8), 20 | 0.955 |

*Supplement table 7: Comparison of recovered and not-recovered respondents with Post-COVID-19 syndrome.*

Abbreviations: 95%CI: 95% Confidence interval; ESS: Epworth Sleepiness Scale; FSS: Fatigue Severity Score; PCR: Polymerase chain reaction; WHO: World Health Organisation.

Statistic: Clinical phenomenology of fatigue and present comorbidities as well as serological findings were analysed using Mann-Whitney U Test (MWU) and Chi-Square-Test, respectively. Adjustment for multiple testing was performed by Bonferroni procedure in regard to each domain independently: patient characteristics: p-value < 0.0125, acute infection: p-value < 0.004, severity of COVID-19 infection: p-value < 0.0125, time variable: p-value < 0.05, fatigue in Post-COVID-19 syndrome: p-value < 0.025, sleep disturbances and mental condition in Post-COVID-19 syndrome: p-value < 0.01; therapy: p-value < 0.006; vaccination: p-value < 0.017

| **Variable** | **Respondents with confirmed infection** | **Respondents without confirmed infection** | **p-value** |
| --- | --- | --- | --- |
| **Patients characteristics** |  |  |  |
| Age, years, mean (95%CI), n | 44.9 (43.4-46.4), 256 | 43.6 (40.5-46.7), 53 | 0.509 |
| Female sex, n (%) | 205/256 (80.1) | 44/53 (83.0) | 0.706 |
| Comorbidities, n (%) | 99/255 (38.8) | 11/53 (20.7) | **0.008** |
| **Symptoms of acute COVID-19, n (%)** |  |  |  |
| Headache | 201/256 (78.5) | 20/53 (37.7) | **0.001** |
| Fever | 133/256 (52.0) | 19/53 (35.8) | 0.035 |
| Dyspnoea | 111/256 (43.4) | 22/53 (41.5) | 0.879 |
| Cough | 150/256 (58.6) | 28/53 (52.8) | 0.449 |
| Cold | 93/256 (36.3) | 13/53 (24.5) | 0.133 |
| Sore throat | 122/256 (47.7) | 22/53 (41.5) | 0.452 |
| Pain | 184/256 (71.8) | 38/53 (71.7) | 1.0 |
| Gastrointestinal symptoms | 56/256 (21.9) | 17/53 (32.1) | 0.114 |
| Fatigue | 227/256 (88.7) | 46/53 (86.8) | 0.644 |
| Sleep disturbance | 88/256 (34.4) | 15/53 (28.3) | 0.428 |
| Skin alteration | 17/256 (6.6) | 9/53 (16.9) | 0.025 |
| Anosmia | 76/256 (29.7) | 12/53 (22.7) | 0.403 |
| **Severity of COVID-19** |  |  |  |
| Hospitalisation, n (%) | 31/256 (12.1) | 2/53 (3F.8) | 0.088 |
| Intubation, n (%) | 26/256 (10.2) | 3/56 (5.7) | 0.439 |
| Oxygen requirement, n (%) | 6/256 (2.3) | 0/53 (0) | 0.595 |
| ICU without intubation, n (%) | 8/256 (3.1) | 0/53 (0) | 0.127 |
| **Time variable** |  |  |  |
| Time between onset of acute infection and questionnaire, months, mean (95%CI), n | 12.3 (11.8-12.7), 256 | 17.4 (15.8-19.0), 53 | **<0.001°** |
| **Fatigue in Post-COVID-19 Syndrome** |  |  |  |
| Fatigue, n (%) | 203/256 (79.3) | 44/53 (83.0) | 0.706 |
| FSS, mean (95%CI), n | 5.8 (5.6-5.9), 203 | 5.5 (5.1-5.9), 44 | 0.208 |
| **Sleep disturbances and mental condition in Post-COVID-19 Syndrome** |  |  |  |
| Sleep disturbances, n (%) | 124/242 (51.2) | 24/52 (46.2) | 0.543 |
| Insomnia Severity Index, mean (95%CI), n | 13.4 (12.5-14.3), 198 | 12.4 (10.5-14.3), 43 | 0.276 |
| ESS, mean (95%CI), n | 11.1 (10.4-11.8), 189 | 10.1 (8.6-11.6), 37 | 0.213 |
| WHO-5 Well-being Index, mean (95%CI), n | 9.1 (8.4-9.8), 197 | 9.1 (7.5-10.8), 42 | 0.956 |
| Sense of comprehension, n (%) | 87/217 (40.1) | 19/49 (38.8) | 1.0 |
| **Therapies** |  |  |  |
| Improvement physiotherapy in %, mean (95%CI) | 31.1 (24.8-37.4), 87 | 33.5 (20.6-46.8), 17 | 0.585 |
| Improvement occupational therapy in %, mean (95%CI), n | 28.0 (20.6-35.8), 53 | 10.0 (-17.8-37.8), 5 | 0.070 |
| Improvement Relaxation methods in %, mean (95%CI) | 37.0 (28.6-45.8), 37 | 30.0 (8.5-51.6), 8 | 0.390 |
| Improvement massage in %, mean (95%CI), n | 30.7 (21.2-40.2), 35 | 33.9 (17.4-50.4), 9 | 0.469 |
| Improvement rehabilitation in %, mean (95%CI), n | 29.6 (22.7-36.5), 43 | 39.0 (2.7-75.3), 5 | 0.497 |
| Improvement antihistaminic medications in %, mean (95%CI), n | 59.1 (49.8-688), 18 | 55.6 (32.1-79.1), 8 | 0.565 |
| Improvement over the time, n (%) | 175/256 (68.4) | 25/53 (47.1) | **0.003** |
| **Vaccination** |  |  |  |
| Vaccination, n (%) | 188/200 (94.0) | 38/50 (76.0) | 0.135 |
| Improvement vaccination in %, mean (95%CI), n | 41.7 (34.5-48.9), 55 | 47.1 (32.1-62.0), 15 | 0.366 |
| Worsening vaccination in %, mean (95%CI), n | 28.7 (22.8-34.6), 32 | 26.0 (22.8-34.6), 14 | 0.932 |

*Supplement table 8: Comparison of respondents with and without confirmed infection Post-COVID-19 syndrome.*

Abbreviations: 95%CI: 95% Confidence interval; ESS: Epworth Sleepiness Scale; FSS: Fatigue Severity Score; PCR: Polymerase chain reaction; WHO: World Health Organisation.

Statistic: Clinical phenomenology of fatigue and present comorbidities as well as serological findings were analysed using Mann-Whitney U Test (MWU) and Chi-Square-Test, respectively. Adjustment for multiple testing was performed by Bonferroni procedure in regard to each domain independently: patient characteristics: p-value < 0.0125, acute infection: p-value < 0.004, severity of COVID-19 infection: p-value < 0.0125, time variable: p-value < 0.05, fatigue in Post-COVID-19 syndrome: p-value < 0.025, sleep disturbances and mental condition in Post-COVID-19 syndrome: p-value < 0.01; therapy: p-value < 0.007; vaccination: p-value < 0.017

Exact p-values given: **°**1.9823E-8

*
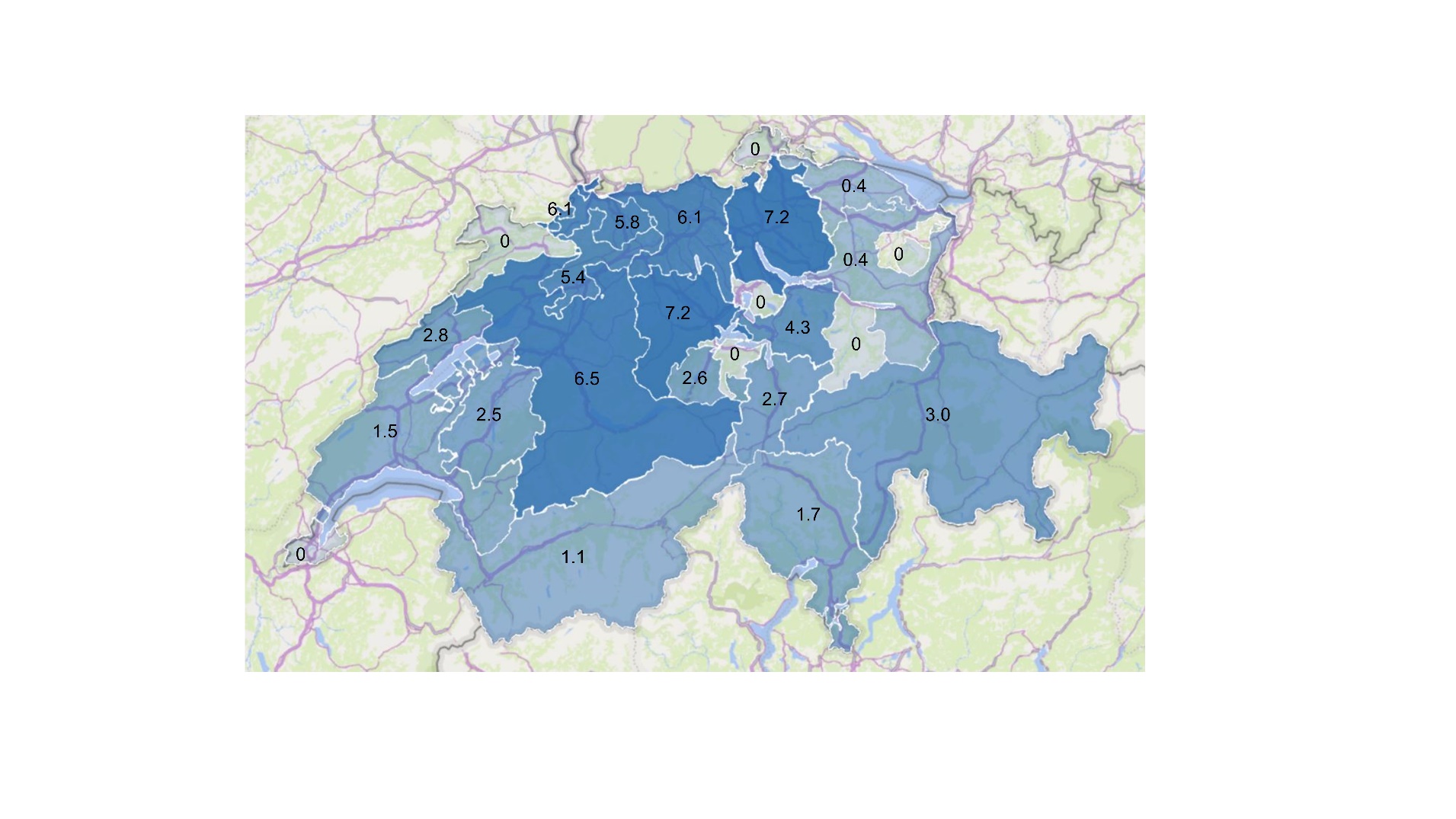
*

*Supplement figure 1: Swiss canton’s distribution of respondent pro 100’000 inhabitants*

Illustrated with Excel Maps 2016
